# An Agmon–Allegretto–Piepenbrink principle for Schrödinger operators

**DOI:** 10.1007/s13398-022-01293-7

**Published:** 2022-07-13

**Authors:** Stefano Buccheri, Luigi Orsina, Augusto C. Ponce

**Affiliations:** 1 Faculty of Mathematics, University of Vienna, Oskar-Morgenstern-Platz 1, 1090 Vienna, Austria; 2 Dipartimento di Matematica, “Sapienza” Università di Roma, P.le A. Moro 2, 00185 Roma, Italy; 3 Université catholique de Louvain, Institut de Recherche en Mathématique et Physique, Chemin du cyclotron 2, L7.01.02, 1348 Louvain-la-Neuve, Belgium

**Keywords:** Schrödinger operator, AAP principle, Poincaré inequality, Singular potential, Primary 35J10, 35R05, 46E35, Secondary 35B05, 35J15, 35J20

## Abstract

We prove that each Borel function $V : \Omega \rightarrow [{-\infty }, +\infty ]$ defined on an open subset $\Omega \subset {\mathbb R}^{N}$ induces a decomposition $\Omega = S \cup \bigcup _{i} D_{i}$ such that every function in $W^{1,2}_{0}(\Omega ) \cap L^{2}(\Omega ; V^{+} \,\mathrm {d}x)$ is zero almost everywhere on *S* and existence of nonnegative supersolutions of $-\Delta + V$ on each component $D_{i}$ yields nonnegativity of the associated quadratic form $\int _{D_{i}} (|\nabla \xi |^2+V\xi ^2).$

## Introduction

Given a smooth bounded connected open set $\Omega \subset \mathbb {R}^{N}$ with $N \ge 2$ and a Borel function $V : \Omega \rightarrow [{-\infty }, +\infty ]$, we investigate the relation between existence of nonnegative solutions of the Dirichlet problem

1.1 $$\begin{aligned} \left\{ \begin{aligned}&- \Delta u + Vu = \mu&\quad \text {in }\Omega ,\\&u = 0&\quad \text {on }\partial \Omega , \end{aligned} \right. \end{aligned}$$

and nonnegativity of the associated quadratic form, namely

1.2 $$\begin{aligned} \int _{\Omega } (|\nabla \xi |^2+V\xi ^2) \ge 0. \end{aligned}$$

When *V* belongs to a Lebesgue space $L^{p}(\Omega )$ with $p > N/2$ or, more generally, to the Kato class $K(\Omega )$, see Definition 2.1 below, the Agmon–Allegretto–Piepenbrink (AAP) principle provides a strong link between existence of nonnegative supersolutions and certain spectral properties of the Schrödinger operator $-\Delta + V$; see e.g. [27]. A typical formulation of the AAP principle for (1.1) is the following

### Theorem 1.1

If $V \in K(\Omega )$, then the Dirichlet problem (1.1) has a nontrivial nonnegative solution $u \in W_{0}^{1, 2}(\Omega )$ for some nonnegative $\mu \in L^{2}(\Omega )$ if and only if inequality (1.2) holds for every $\xi \in W_{0}^{1, 2}(\Omega )$.

Such a statement is known to specialists but in Sect. 2 we present a proof for the reader’s convenience. A key ingredient is the compact imbedding $W_{0}^{1, 2}(\Omega ) \subset L^{2}(\Omega ; V \,\mathrm {d}x)$ that holds when $V \in K(\Omega )$.

Various attempts have been made to further generalize the class of potentials *V* in the AAP principle; see for instance [13–15, 21]. However, an equivalence as in Theorem 1.1 for a general Borel function *V* is hopelessly false. For example, taking $\Omega = B_{1}$ to be the unit ball centered at 0 and $V(x) = 1/|x_{1}|^{\alpha }$ with $1 \le \alpha < 2$, one has that (1.2) is trivially satisfied by positivity of *V* but there exists no nonnegative function *v* that satisfies $-\Delta v + V v \ge 0$ in the sense of distributions in $\Omega$, other than the trivial one; see [17, Theorem 1.4] and Example 5.5 below.

In contrast, existence of nonnegative supersolutions typically yields nonnegativity of the quadratic form under weaker assumptions on *V*, as it has been proved in [15] for $V \in L^{1}(\Omega )$. As we shall see, this property is true even for general Borel potentials *V*.

One of the novelties of our work is to address this issue by adapting the concept of supersolution to the Schrödinger operator $- \Delta + V$. Our approach is based on the existence of a Borel set $S \subset \Omega$ with the following properties:

- every solution *u* of (1.1) satisfies $u = 0$ almost everywhere in *S*,
- if moreover *u* is nonnegative, then $\mu \le 0$ on *S*, regardless of $\mu$ on $\Omega \setminus S$.

The Borel set *S* that we consider is related solely to the positive part $V^{+}$ and has been introduced in [19] as the level set1.3where $\widehat{\zeta _{1}}$ denotes the precise representative of the *torsion function* $\zeta _{1}$ , which minimizes the energy functional

$$\begin{aligned} \xi \in W_{0}^{1, 2}(\Omega ) \cap L^{2}(\Omega ; V^{+} \,\mathrm {d}x) \longmapsto \frac{1}{2} \int _{\Omega } (|\nabla \xi |^{2} + V^{+}\xi ^{2}) - \int _{\Omega } \xi . \end{aligned}$$

One verifies that $\widehat{\zeta _{1}}$ exists at every point of $\Omega$ since $\zeta _{1}$ can be written as the difference between a continuous and a bounded subharmonic function; see e.g. [18, Proposition 8.1]. This set *S* has been used in [19] to investigate the failure of the strong maximum principle for the Schrödinger operator $- \Delta + V^{+}$ and non-existence of solutions of the associated Dirichlet problem. That *S* defined by (1.3) satisfies properties (a) and (b) is justified in Sect. 5. An alternative way of identifying *S* without explicitly relying on the torsion function is also available using the Wiener criterion of Dal Maso and Mosco [6]; see [24].

In a classical situation where $V^{+} \in K(\Omega )$, the strong maximum principle holds and then one has $S = \emptyset$. However, for general potentials $V^+$ one should expect to have $S \ne \emptyset$. For example, if $a \in \Omega$ and $V(x) = 1/|x - a|^{2}$, then $S = \{a\}$. The set *S* may be larger than a finite union of singletons: Take a smooth open set $\omega \Subset \Omega$ and $V(x) = 1/d(x, \partial \omega )^{2}$, then $S = \partial \omega$. Choosing instead $V(x) = 1/d(x, \omega )^{2}$, one has $S = {\overline{\omega }}$. These two examples are physically related to the localized effect of particles in Quantum Mechanics that has been beautifully addressed in recent years by I. Díaz [9, 10].

From [19], we know that $\Omega \setminus S$ can be written as a disjoint union of its Sobolev-connected components, namely

1.4 $$\begin{aligned} \Omega \setminus S = \bigcup _{i \in I}{D_{i}}\,, \end{aligned}$$

where the index set *I* is finite or countably infinite. In the terminology introduced in [19, Sections 10 and 11], each set $D_{i}$ is Sobolev-open in the sense that there exists $v_{i} \in W_{0}^{1, 2}(\Omega )$ whose precise representative $\widehat{v_{i}}$ is defined everywhere in $\Omega$ and $D_{i} = \{\widehat{v_{i}} > 0\}$. In addition, $D_{i}$ is Sobolev-connected meaning that it cannot be further written as a disjoint finite reunion of Sobolev-open subsets. When *S* is closed in $\Omega$ with respect to the Euclidean topology, the sets $D_{i}$ coincide with the usual connected components of $\Omega \setminus S$.

Decomposition (1.4) propagates to every $\xi \in W_{0}^{1, 2}(\Omega ) \cap L^{2}(\Omega ; V^{+} \,\mathrm {d}x)$ as we prove in Sect. 4 that $\xi = 0$ almost everywhere in *S* and, for every $i \in I$, $\xi \chi _{D_{i}} \in W_{0}^{1, 2}(\Omega )$ ; see Proposition 4.3. Thus, $\xi$ can be written as a sum of Sobolev functions,

1.5 $$\begin{aligned} \xi = \sum _{i \in I}{\xi \chi _{D_{i}}} \quad \text {almost everywhere in }\Omega . \end{aligned}$$

In the spirit of the decompositions (1.4) and (1.5), we localize the AAP principle each component $D_i$ :

### Theorem 1.2

Let $i \in I$. If (1.1) has a nonnegative distributional solution *u* for a finite Borel measure $\mu$ in $\Omega$ such that $\mu \ge 0$ in $D_{i}$ and $\int _{D_{i}} u > 0$, then

1.6 $$\begin{aligned} \int _{D_{i}} (|\nabla \xi |^{2} + V\xi ^{2}) \ge 0 \quad \text {for every }\xi \in W_{0}^{1, 2}(\Omega ) \cap L^{2}(\Omega ; V^{+} \,\mathrm {d}x). \end{aligned}$$

We recall that *u* is a distributional solution of (1.1) whenever $u \in W^{1, 1}_0(\Omega ) \cap L^1(\Omega ; V \,\mathrm {d}x)$ and

1.7 $$\begin{aligned} -\Delta u + V u = \mu \quad \text {in the sense of distributions in }\Omega . \end{aligned}$$

We show in Sects. 4 and 5 that this equation can be localized in $D_{i}$ so that $u\chi _{D_{i}}$ satisfies

$$\begin{aligned} -\Delta (u\chi _{D_{i}}) + V u\chi _{D_{i}} = \mu \lfloor _{D_{i}}{} - \tau \quad \text {in the sense of distributions in }\Omega , \end{aligned}$$

where the measure $\mu \lfloor _{D_{i}}$ is defined by $\mu \lfloor _{D_{i}}(A) = \mu (D_{i} \cap A)$ for every Borel set $A \subset \Omega$ and $\tau$ is a measure carried by *S*, that is $|\tau |(\Omega \setminus S) = 0$. Under the assumptions of Theorem 1.2, we have that $\tau$ is nonnegative and we may apply the strong maximum principle for $-\Delta + V^{+}$ in $D_{i}$ to deduce thatwhere  is the average integral on the ball $B_{r}(x)$.

Theorem 1.2 thus provides one with a Poincaré inequality that gives the imbedding

$$\begin{aligned} W_{0}^{1, 2}(\Omega ) \cap L^{2}(\Omega ; V^{+} \,\mathrm {d}x) \subset L^{2}(D_{i}; V^{-} \,\mathrm {d}x), \end{aligned}$$

which can be seen as a compatibility condition between $V^+$ and $V^-$ to have existence of supersolutions in $D_{i}$ . When (1.6) is satisfied, we consider the vector spaceequipped with the seminormWe then denote by $\mathcal {H}_{i}(\Omega )$ the completion of $H_{i}(\Omega )$.

In the spirit of the concept of weighted spectral gap by Pinchover and Tintarev [22], it is natural to investigate whether there is room for an improvement of (1.6) in the form

1.8 $$\begin{aligned} \int _{D_{i}} (|\nabla \xi |^2+V\xi ^2) \ge \int _{D_{i}} w_{i} \xi ^2{} \quad \text {for every }\xi \in W_{0}^{1, 2}(\Omega ) \cap L^{2}(\Omega ; V^{+} \,\mathrm {d}x), \end{aligned}$$

for some measurable weight $w_{i} : D_{i} \rightarrow (0, +\infty )$. In contrast with [22], we do not require $w_{i}$ to be continuous. An inequality of the type (1.8) is typically used to prove decay of solutions associated to the operator $-\Delta + V$ and long-time behavior of the $L^p$ norm for the Schrödinger semigroup in unbounded domains; see [1, 20, 26]. For the critical Hardy potential $V = -\kappa /|x|^{2}$, estimate (1.8) holds with $w_{i}$ constant and such an inequality has been used to investigate nonlinear eigenvalue problems and asymptotics of the heat equation; see [5, 8, 11, 28].

### Theorem 1.3

Let $i \in I$. If (1.1) has a nonnegative distributional solution for a finite Borel measure $\mu$ in $\Omega$ such that $\mu \ge 0$ in $D_{i}$ and $\mu (D_{i}) > 0$, then there exists a measurable function $w_{i} : D_{i} \rightarrow (0, +\infty )$ such that (1.8) holds.

We prove Theorem 1.3 in Sect. 8. The possibility that the measure in the statement of Theorem 1.2 satisfies $\mu (D_{i}) = 0$ does not exclude the existence of another one with $\widetilde{\mu }\ge 0$ in $D_{i}$ and $\widetilde{\mu }(D_{i}) > 0$, for which a nonnegative solution exists and then the stronger conclusion of Theorem 1.3 holds; see Example 7.7. This phenomenon is due to the fact that distributional solutions need not belong to $L^{2}(\Omega ; V^{-} \,\mathrm {d}x)$, see Corollary 8.2, and is in sharp contrast to what happens with the operator $-\Delta - \lambda _{1}$, where $\lambda _{1}$ is the first eigenvalue of the Laplacian. In such a case, one has $S = \emptyset$ and if *u* solves

$$\begin{aligned} \left\{ \begin{aligned}&- \Delta u - \lambda _{1}u = \mu&\quad \text {in }\Omega ,\\&u = 0&\quad \text {on }\partial \Omega , \end{aligned} \right. {} \end{aligned}$$

for some finite nonnegative measure $\mu$, then using the first eigenfunction $\varphi$ of $-\Delta$ as test function one deduces that necessarily $\mu = 0$ and $u = \alpha \varphi$ with $\alpha \in {\mathbb R}$.

Under the stronger assumption (1.8), any element $\xi \in \mathcal {H}_i(\Omega )$ can be naturally associated to a function in $L^2(D_i; w_i \,\mathrm {d}x)$ and in particular is well-defined almost everywhere in $D_{i}$ . Moreover, using the direct method of the Calculus of Variations one easily deduces that, for every $h \in L^{2}(D_{i}; w_{i} \,\mathrm {d}x)$, there exists a unique minimizer $\theta _{i, h}$ of the energy functional

1.9 $$\begin{aligned} E(\xi ) = \frac{1}{2} \Vert \xi \Vert _{i}^{2} - \int _{D_{i}} w_{i}h\xi {} \quad \text {with }\xi \in \mathcal {H}_{i}(\Omega ). \end{aligned}$$

While $\theta _{i, h}$ satisfies the Euler-Lagrange equation in $\mathcal {H}_{i}(\Omega )$, it need not be true that $\theta _{i, h}$ verifies (1.1) in the sense of distributions for some finite measure $\mu$; see Example 9.1. We prove nevertheless in Sect. 9 the following

### Theorem 1.4

Let $i \in I$. If (1.1) has a nonnegative distributional solution *u* for a finite Borel measure $\mu$ in $\Omega$ such that $\mu \ge 0$ in $D_{i}$ and $\mu (D_{i}) > 0$, then there exists a bounded measurable function $w_{i} : D_{i} \rightarrow (0, +\infty )$ satisfying (1.8) and such that, for every $h \in L^{2}(D_{i}; w_{i} \,\mathrm {d}x)$ with $0 \le h \le u$, the minimizer of (1.9) is a nonnegative distributional solution of (1.1) for a finite Borel measure $\mu _{i,h}$ in $\Omega$ that verifies

$$\begin{aligned} \mu _{i, h} = w_{i} h \,\mathrm {d}x \quad \text {on }D_{i}\,. \end{aligned}$$

An interesting open problem is to find a weight $w_{i}$ such that the conclusion of Theorem 1.4 holds for every nonnegative $h \in L^{2}(D_{i}; w_{i} \,\mathrm {d}x)$.

## Proof of Theorem 1.1

In this section we provide a proof of Theorem 1.1. We assume that $N \ge 3$ ; the case $N = 2$ requires some adaptation as one needs to modify the definition of the Kato class using the logarithm in replacement of the potential $1/|z|^{N-2}$. Let us start recalling the definition of the Kato class $K(\Omega )$.

### Definition 2.1

A Borel function $V:\Omega \rightarrow [{-\infty }, +\infty ]$ belongs to the Kato class $K(\Omega )$ whenever

$$\begin{aligned} \lim _{r\rightarrow 0}{\sup _{x \in \Omega } \int _{B_{r}(x)\cap \Omega }\frac{|V(y)|}{|x-y|^{N-2}} \,\mathrm {d}y} = 0. \end{aligned}$$

The proof of Theorem 1.1 is based on the following lemma:

### Lemma 2.2

If $V\in K(\Omega )$, then for each $\epsilon >0$ there exists $C > 0$ such that

$$\begin{aligned} \int _{\Omega } |V|\varphi ^2{} \le \epsilon \int _{\Omega }|\nabla \varphi |^2 + C \int _{\Omega }\varphi ^2 \quad \text {for every } \varphi \in C^\infty _c(\Omega ). \end{aligned}$$

To our knowledge, the proof of this lemma goes back to [25, Chapter 6, Theorem 9.3]. We provide a more direct proof based on [29]. The estimate above implies that $W_{0}^{1, 2}(\Omega ) \subset L^{2}(\Omega ; V \,\mathrm {d}x)$ and that the inclusion is compact. Indeed, by density of $C_{c}^{\infty }(\Omega )$ in $W_{0}^{1, 2}(\Omega )$, the inequality holds with $\varphi = \xi \in W_{0}^{1, 2}(\Omega )$. Moreover, if $(\xi _{n})_{n \in {\mathbb N}}$ is a bounded sequence in $W_{0}^{1, 2}(\Omega )$, then by the Rellich-Kondrashov Compactness Theorem we may extract a subsequence such that $\xi _{n_{j}} \rightarrow u$ in $L^{2}(\Omega )$ for some $u \in W_{0}^{1, 2}(\Omega )$. Using the inequality above for $\xi _{n_{j}} - u$ we then have, for every $\epsilon > 0$,

$$\begin{aligned} \limsup _{j \rightarrow \infty }{\int _{\Omega } |V|(\xi _{n_{j}} - u)^2} \le \epsilon \limsup _{j \rightarrow \infty }\int _{\Omega }|\nabla (\xi _{n_{j}} - u)|^2. \end{aligned}$$

Since $\epsilon$ is arbitrary, the convergence $\xi _{n_{j}} \rightarrow u$ in $L^{2}(\Omega ; V \,\mathrm {d}x)$ then follows.

### Proof of Lemma 2.2

We first show that, for every $\varphi \in C_c^\infty (\Omega )$,

2.1 $$\begin{aligned} \int _{\Omega }|V| \varphi ^2{} \le \biggl ( \sup _{x \in \Omega } \int _{{{\,\mathrm{supp}\,}}{\varphi }} \frac{|V(y)|}{|x-y|^{N-2}} \,\mathrm {d}y \biggr ) \int _{\Omega }|\nabla \varphi |^2. \end{aligned}$$

This inequality is proved in [25, Chapter 6, Theorem 8.8] using Bessel potentials, but here we follow a simpler idea from [29]: Since

$$\begin{aligned} |\varphi (x)|{} \le {{C}_1} \int _{\Omega }\frac{|\nabla \varphi (y)|}{|x-y|^{N-1}} \,\mathrm {d}y, \end{aligned}$$

we get

2.2 $$\begin{aligned} \int _{\Omega }|V(x)| |\varphi (x)|^{2} \,\mathrm {d}x&\le {{C}_1} \int _{\Omega }\left( \int _{\Omega }\frac{|V(x)| |\varphi (x)|}{|x-y|^{N-1}} \,\mathrm {d}x\right) |\nabla \varphi (y)| \,\mathrm {d}y\nonumber \\&\le {{C}_1} \biggl (\int _{\Omega }\left( \int _{\Omega } \frac{|V(x)| |\varphi (x)|}{|x-y|^{N-1}} \,\mathrm {d}x \right) ^2 \,\mathrm {d}y\biggr )^{\frac{1}{2}} \left( \int _{\Omega }|\nabla \varphi (y)|^2 \,\mathrm {d}y\right) ^{\frac{1}{2}}.\nonumber \\ \end{aligned}$$

Note that

$$\begin{aligned} \left( \int _{\Omega } \frac{|V(x)| |\varphi (x)|}{|x-y|^{N-1}} \,\mathrm {d}x \right) ^2{} \le \left( \int _{{{\,\mathrm{supp}\,}}{\varphi }} \frac{|V(z)|}{|z-y|^{N-1}} \,\mathrm {d}z\right) \left( \int _{\Omega }\frac{|V(x)| |\varphi (x)|^2}{|x-y|^{N-1}} \,\mathrm {d}x\right) {} \end{aligned}$$

and

$$\begin{aligned} \int _{\Omega } \frac{\,\mathrm {d}y}{|z-y|^{N-1}|x-y|^{N-1}} \le \frac{C_2}{|z-x|^{N-2}}. \end{aligned}$$

From both estimates and Fubini’s theorem, we get

$$\begin{aligned} \begin{aligned} \int _{\Omega }\biggl (\int _{\Omega }\frac{|V(x)| |\varphi (x)|}{|x-y|^{N-1}} \,\mathrm {d}x \biggr )^2 \,\mathrm {d}y&\le {C_2} \int _{{{\,\mathrm{supp}\,}}{\varphi }}\int _{\Omega } \frac{|V(z)||V(x)| |\varphi (x)|^{2}}{|z-x|^{N-2}} \,\mathrm {d}x \,\mathrm {d}z\\&\le {C_2} \biggl (\sup _{x\in \Omega }\int _{{{\,\mathrm{supp}\,}}{\varphi }} \frac{|V(z)|}{|z-x|^{N-2}} \,\mathrm {d}z \biggr ) \int _{\Omega } |V(x)||\varphi (x)|^2 \,\mathrm {d}x. \end{aligned} \end{aligned}$$

Combining this estimate with (2.2), we obtain (2.1).

To conclude, we cover $\Omega$ with a regular net of balls $B_{{\delta }}(x_i)$ with $i=1,\dots , M$, where the number of overlaps depends solely on the dimension *N*. Let $\psi _1, \dots , \psi _M$ be a smooth partition of unity subordinated to this covering. Given $\varphi \in C_{c}^{\infty }(\Omega )$, we have

2.3 $$\begin{aligned} \int _{\Omega } |V|\varphi ^2{} \le {C_3} \sum _{i=1}^M \int _{\Omega } |V|(\varphi \psi _i)^2, \end{aligned}$$

for some constant ${C_3} > 0$ depending on *N*. By (2.1) applied to $\varphi \psi _{i}$, we get

2.4 $$\begin{aligned} \int _{\Omega }|V| (\varphi \psi _{i})^2 \le \eta (\delta ) \int _{\Omega }|\nabla (\varphi \psi _{i})|^2, \end{aligned}$$

withWe have $0 \le \psi _{i} \le 1$ and we may assume that $|\nabla \psi _{i}| \le C_4/\delta$ for every *i*. Combining (2.3) and (2.4), we get

$$\begin{aligned} \int _{\Omega } |V|\varphi ^2 \le {C_5}\eta (\delta ) \biggl (\int _{\Omega }|\nabla \varphi |^2 + \frac{1}{\delta ^{2}} \int _{\Omega }\varphi ^2\biggr ), \end{aligned}$$

where ${C_5} > 0$ depends on *N*. Since $V \in K(\Omega )$, we have $\eta (\delta ) \rightarrow 0$ as $\delta \rightarrow 0$. Thus, given $\epsilon > 0$, we may take $\delta > 0$ small enough so that ${C_5}\eta (\delta ) \le \epsilon$. $\square$

*Proof of Theorem *1.1 “$\Longleftarrow$” We prove the reverse implication, so we assume that

2.5 $$\begin{aligned} \int _{\Omega }(|\nabla \xi |^2 + V\xi ^2){} \ge 0 \quad \text {for every } \xi \in W^{1,2}_0(\Omega ). \end{aligned}$$

Our goal is to prove that the minimization problem

2.6 $$\begin{aligned} \lambda _1{} =\inf \left\{ \int _{\Omega }(|\nabla \xi |^2+V\xi ^2) \ : \ \xi \in W^{1,2}_0(\Omega ) \ \text{ and } \ \int _{\Omega } \xi ^{2} =1\right\} \end{aligned}$$

achieves its infimum for some nonnegative function *u*. Note that, thanks to (2.5), the infimum $\lambda _1$ is well-defined and nonnegative. Once one knows that the minimum exists, it is standard to show that

$$\begin{aligned} -\Delta u + Vu = \lambda _1 u \quad \text {in the sense of distributions in }\Omega . \end{aligned}$$

Take a minimizing sequence $(\xi _{n})_{n \in {\mathbb N}}$ of (2.6). We show that $(\nabla \xi _{n})_{n \in {\mathbb N}}$ is bounded in $L^{2}(\Omega ; {\mathbb R}^{N})$. To this end, for *n* large enough, we apply Lemma 2.2 with $\epsilon = 1/2$ to get

$$\begin{aligned} \int _{\Omega }|\nabla \xi _n|^2{} \le (\lambda _1 + 1) - \int _{\Omega }V\xi _n^2{} \le (\lambda _1 + 1) + \frac{1}{2} \int _{\Omega }|\nabla \xi _n|^2 + C \int _{\Omega }\xi _n^2. \end{aligned}$$

Recalling the normalization condition of $\xi _n$ , it follows that

$$\begin{aligned} \frac{1}{2} \int _{\Omega }|\nabla \xi _n|^2 \le (\lambda _1 + 1) + C. \end{aligned}$$

By boundedness of $(\xi _{n})_{n \in {\mathbb N}}$ in $W_{0}^{1, 2}(\Omega )$ and compactness of the inclusion $W_{0}^{1, 2}(\Omega ) \subset L^{2}(\Omega ; V \,\mathrm {d}x)$, we may extract a subsequence $(\xi _{n_{j}})_{j \in {\mathbb N}}$ that converges weakly to some *v* in $W^{1, 2}_0(\Omega )$ and also strongly in $L^2(\Omega )$ and $L^{2}(\Omega ; V \,\mathrm {d}x)$. We thus have

$$\begin{aligned} \int _{\Omega }(|\nabla v|^2 + Vv^2) \le \liminf _{j \rightarrow \infty }\int _{\Omega }(|\nabla \xi _{n_{j}}|^2 + V\xi _{n_{j}}^2)=\lambda _1, \end{aligned}$$

with $\int _{\Omega } v^{2} =1$. We deduce that *v* is a minimizer of (2.6). Hence, the nonnegative function |*v*| is also a minimizer and we have the conclusion by taking $u = |v|$ and $\mu = \lambda _{1}u$. $\square$

To prove the direct implication of Theorem 1.1, we show that existence of a positive supersolution yields a weighted Poincaré inequality. The following lemma is based on a typical variational argument that can be implemented even for a general Borel function *V*.

### Lemma 2.3

Let $f \in L^{2}(\Omega )$ and $u \in W_{0}^{1, 2}(\Omega ) \cap L^{2}(\Omega ; V \,\mathrm {d}x)$ be nonnegative functions such that, for every nonnegative $\xi \in W_{0}^{1, 2}(\Omega ) \cap L^{2}(\Omega ; V \,\mathrm {d}x)$,

2.7 $$\begin{aligned} \int _{\Omega }(\nabla u \cdot \nabla \xi + V u \xi ){} \ge \int _{\Omega } f \xi . \end{aligned}$$

Then, for every $\xi \in W_{0}^{1, 2}(\Omega ) \cap L^{2}(\Omega ; V \,\mathrm {d}x)$, we have $f\xi ^{2} = 0$ almost everywhere in $\{u = 0\}$ and

2.8 $$\begin{aligned} \int _{\{u> 0\}} (|\nabla \xi |^{2} + V \xi ^{2}) \ge \int _{\{u > 0\}} \frac{f}{u} \xi ^{2}. \end{aligned}$$

### Proof of Lemma 2.3

Given $\xi \in W_{0}^{1, 2}(\Omega ) \cap L^{2}(\Omega ; V \,\mathrm {d}x) \cap L^{\infty }(\Omega )$ and $\epsilon > 0$, we have  and $\eta$ is nonnegative. Using $\eta$ as a test function in (2.7), we get

$$\begin{aligned} 2\int _{\Omega } \frac{\nabla u \cdot \nabla \xi }{u + \epsilon } \xi - \int _{\Omega } \frac{|\nabla u|^2 }{(u + \epsilon )^2}\xi ^2 + \int _{\Omega }\frac{V u }{u + \epsilon }\xi ^2 \ge \int _{\Omega } \frac{f}{u + \epsilon } \xi ^{2}. \end{aligned}$$

Since $\nabla u = 0$ almost everywhere in $\{u = 0\}$,

$$\begin{aligned} 2\frac{\nabla u \cdot \nabla \xi }{u + \epsilon } \xi {} \le \frac{|\nabla u|^2 }{(u + \epsilon )^2}\xi ^2 + |\nabla \xi |^{2} \chi _{\{u > 0\}}. \end{aligned}$$

Thus,

2.9 $$\begin{aligned} \int _{\{u > 0\}} |\nabla \xi |^{2} + \int _{\Omega } \frac{V u }{u + \epsilon }\xi ^2 \ge \int _{\Omega } \frac{f}{u + \epsilon } \xi ^2. \end{aligned}$$

Note that, as $\epsilon \rightarrow 0$,

$$\begin{aligned} \frac{V u }{u + \epsilon }\xi ^2 \rightarrow V\xi ^2 \chi _{\{u> 0\}} \quad \text {and} \quad {} \frac{f}{u + \epsilon }\xi ^2 \rightarrow \frac{f}{u}\xi ^2 \chi _{\{u> 0\}} +\infty \,\chi _{\{f\xi ^{2} > 0\} \cap \{u = 0\}}. \end{aligned}$$

Since

$$\begin{aligned} \Bigl | \frac{V u }{u + \epsilon }\xi ^2 \Bigr |{} \le |V|\xi ^{2} \in L^{1}(\Omega ) \end{aligned}$$

it follows from Fatou’s lemma and (2.9) that $\{f\xi ^{2} > 0\} \cap \{u = 0\}$ is negligible with respect to the Lebesgue measure. Applying the Dominated Convergence Theorem, we deduce estimate (2.8) under the additional assumption that $\xi \in L^{\infty }(\Omega )$. To get the estimate for a general $\xi \in W_{0}^{1, 2}(\Omega ) \cap L^{2}(\Omega ; V \,\mathrm {d}x)$, it suffices to apply for the truncated function  with $k \ge 0$ and let $k \rightarrow \infty$. $\square$

*Proof of Theorem* 1.1 “$\Longrightarrow$” We now assume that $u \in W_{0}^{1, 2}(\Omega )$ is a nonnegative function that satisfies

$$\begin{aligned} -\Delta u + Vu = \mu {} \quad \text {in the sense of distributions in } \Omega , \end{aligned}$$

where $\mu \in L^{2}(\Omega )$ is also nonnegative. Since $V \in K(\Omega )$, by Lemma 2.2 we have $W_{0}^{1, 2}(\Omega ) \subset L^{2}(\Omega ; V \,\mathrm {d}x)$. As $\mu \in L^{2}(\Omega )$, it then follows from the equation satisfied by *u* and the density of $C_{c}^{\infty }(\Omega )$ in $W_{0}^{1, 2}(\Omega )$,

$$\begin{aligned} \int _{\Omega } (\nabla u \cdot \nabla \xi + Vu\xi {}) = \int _{\Omega } \mu \xi {} \quad \text {for every }\xi \in W_{0}^{1, 2}(\Omega ). \end{aligned}$$

Thus, by Lemma 2.3,

2.10 $$\begin{aligned} \int _{\{u> 0\}} (|\nabla \xi |^{2} + V \xi ^{2}) \ge \int _{\{u > 0\}} \frac{\mu }{u} \xi ^{2} \ge 0 \quad \text {for every }\xi \in W_{0}^{1, 2}(\Omega ). \end{aligned}$$

Since $V \in L^{1}(\Omega )$ and *u* is a nontrivial nonnegative function such that

$$\begin{aligned} \int _{\Omega } (\nabla u \cdot \nabla \xi + V^{+}u\xi {}) \ge 0 \quad \text {for every nonnegative }\xi \in W_{0}^{1, 2}(\Omega ), \end{aligned}$$

by the strong maximum principle, see [23, Proposition 22.2], we have $u > 0$ almost everywhere in $\Omega$ and then (2.10) gives

$$\begin{aligned} \int _{\Omega } (|\nabla \xi |^{2} + V \xi ^{2}) \ge 0 \quad \text {for every }\xi \in W_{0}^{1, 2}(\Omega ). \end{aligned}$$

$$\square $$

## Duality solutions for nonnegative potentials

For each $f \in L^{\infty }(\Omega )$, let $\zeta _{f}$ be the unique minimizer of the energy functional

3.1 $$\begin{aligned} \xi \in W^{1, 2}_0(\Omega )\cap L^2(\Omega ; V^+ \,\mathrm {d}x) \longmapsto \frac{1}{2} \int _{\Omega }(|\nabla \xi |^2+ V^+ \xi ^2) - \int _{\Omega }f \xi . \end{aligned}$$

Denoting by $\mathcal {M}(\Omega )$ the vector space of finite Borel measures in $\Omega$, we recall the following notion from [16]:

### Definition 3.1

Given $\nu \in \mathcal {M}(\Omega )$, a function $u \in L^{1}(\Omega )$ is a *duality solution* of

3.2 $$\begin{aligned} \left\{ \begin{alignedat}{2}&-\Delta u+ V^{+} u = \nu&\quad \text {in } \Omega ,\\&u = 0&\quad \text {on } \partial \Omega , \end{alignedat} \right. \end{aligned}$$

whenever

3.3 $$\begin{aligned} \int _{\Omega }u f = \int _{\Omega } \widehat{\zeta _f} \,\mathrm {d}\nu \quad \text {for every } f \in L^{\infty }(\Omega ). \end{aligned}$$

One verifies that the precise representative $\widehat{\zeta _f}$ is defined at every point in $\Omega$, that is for every $x \in \Omega$ there exists $\widehat{\zeta _{f}}(x) \in {\mathbb R}$ such thatHence, the integral in the right-hand side of (3.3) is well-defined.

Every distributional solution is a duality solution, with the same datum. The converse however need not be true; see Example 5.5. A major advantage of dealing with duality solutions is that, in contrast with distributional solutions, they always exist for every $\nu \in \mathcal {M}(\Omega )$. For example, if $g \in L^{\infty }(\Omega )$, then a combination of the Euler–Lagrange equations satisfied by $\zeta _{f}$ and $\zeta _{g}$ implies that

3.4 $$\begin{aligned} \int _{\Omega } \zeta _{g} f = \int _{\Omega } \zeta _{f}\, g \quad \text {for every } f \in L^{\infty }(\Omega ). \end{aligned}$$

Since $\zeta _{f} = \widehat{\zeta _{f}}$ almost everywhere in $\Omega$ by Lebesgue’s differentiation theorem, we deduce that $\zeta _{g}$ is the duality solution of (3.2) with datum $\nu = g \,\mathrm {d}x$. Moreover, from the estimate

$$\begin{aligned} |\zeta _{g} | \le \Vert g \Vert _{L^{\infty }(\Omega )} \zeta _{1}\,,{} \end{aligned}$$

for every $g \in L^{\infty }(\Omega )$ we then have

3.5 $$\begin{aligned} \widehat{\zeta _{g}} = 0 \quad \text {in }S. \end{aligned}$$

We investigate in this section a counterpart of (3.5) that is valid for any duality solution of (3.2). We begin with the following general property:

### Proposition 3.2

If *u* is a duality solution of (3.2) with datum $\nu \in \mathcal {M}(\Omega )$, then $u = 0$ almost everywhere in *S*.

As we have pointed out, Proposition 3.2 is straightforward when $u = \zeta _{g}$ with $g \in L^{\infty }(\Omega )$. To prove the proposition in full generality, we use the property that, for every $\nu \in \mathcal {M}(\Omega )$, the duality solution *u* of (3.2) satisfies the representation formula

3.6 $$\begin{aligned} u(x) = \int _{\Omega } \widehat{G_x} \,\mathrm {d}\nu \quad {} \text {for almost every }x \in \Omega , \end{aligned}$$

where $G_x$ denotes the duality solution of

$$\begin{aligned} \left\{ \begin{aligned}&-\Delta G_{x}+ V^{+} G_{x} = \delta _{x}&\quad \text {in } \Omega ,\\&G_{x} = 0&\quad \text {on } \partial \Omega . \end{aligned} \right. \end{aligned}$$

Formula (3.6) is proved in [19, Lemma 13.2] when $\nu$ is nonnegative. For a general signed measure $\nu$, one first applies (3.6) to the duality solutions of (3.2) with nonnegative data $\nu ^{+}$ and $\nu ^{-}$. We recall that this step can be performed since duality solutions exist for any data in $\mathcal {M}(\Omega )$. It then follows from (3.6) applied to $\nu ^{+}$ and $\nu ^{-}$ that

$$\begin{aligned} \int _{\Omega } \widehat{G_x} \,\mathrm {d}|\nu | < +\infty \quad {} \text {for almost every }x \in \Omega \end{aligned}$$

and, taking differences, we deduce (3.6) for $\nu = \nu ^{+} - \nu ^{-}$.

### Proof of Proposition 3.2

Since $G_{x}$ is a duality solution, using $\zeta _{1}$ as test function we have

$$\begin{aligned} \int _{\Omega } G_{x} = \int _{\Omega } \widehat{\zeta _{1}} \,\mathrm {d}\delta _{x} = \widehat{\zeta _{1}}(x)\,, \end{aligned}$$

for every $x \in \Omega$. Then, by nonnegativity of $G_{x}$ we deduce that

3.7 $$\begin{aligned} \widehat{G_{x}} = 0 \quad \text {for every }x \in S. \end{aligned}$$

From (3.6), we deduce that $u(x) = 0$ for almost every $x \in S$. $\square$

We now prove a sharper version of Proposition 3.2, where the Lebesgue measure is replaced by the $W^{1, 2}$ capacity. More precisely, we recall that every duality solution *u* has a precise representative $\widehat{u}$ which is defined quasi-everywhere, that is except in a set of $W^{1, 2}$ capacity zero. This is a consequence of the fact that $\Delta u$ is a finite measure in $\Omega$; see [19, Proposition 4.1] and [23, Proposition 8.9]. We then have the following statement whose proof follows along the lines of [19, Proposition 13.3]:

### Proposition 3.3

If *u* is a duality solution of (3.2) with datum $\nu \in \mathcal {M}(\Omega )$, then $\widehat{u} = 0$ quasi-everywhere in *S*.

Let us denote by *F* the fundamental solution of $-\Delta$ and focus our discussion on dimension $N \ge 3$. The starting observation is that if $\nu \in \mathcal {M}(\Omega )$ is extended as 0 outside $\Omega$, then for any smooth bounded open set $U \supset \Omega$ the distributional solution of

$$\begin{aligned} \left\{ \begin{aligned}&-\Delta w = \nu&\quad \text {in } U,\\&w = 0&\quad \text {on } \partial U, \end{aligned} \right. \end{aligned}$$

satisfies $|w| \le F * |\nu |$ almost everywhere in *U*. Hence, if $F * |\nu |$ is bounded, then so is *w* and, by interpolation, we have $w \in W_{0}^{1, 2}(U)$. As a result, $\nu$ belongs to the dual space $(W_{0}^{1, 2}(U))'$ and is *diffuse* with respect to the $W^{1, 2}$ capacity, that is, for any Borel set $A \subset \Omega$ with $W^{1, 2}$ capacity zero, one has $\nu (A) = 0$. The action of $\nu$ as an element in $(W_{0}^{1, 2}(U))'$ is given by3.8In dimension $N = 2$, the strategy above needs to be slightly adapted since $F(x) \rightarrow -\infty$ as $|x| \rightarrow \infty$ and one cannot expect to have $F * |\nu |$ bounded in ${\mathbb R}^{2}$.

### Proof of Proposition 3.3

We first assume that the Newtonian potential $F * |\nu |$ is bounded. In this case, by comparison with $F * |\nu |$ we have $u \in L^{\infty }(\Omega )$ and then $u \in W_{0}^{1, 2}(\Omega ) \cap L^{2}(\Omega ; V^{+} \,\mathrm {d}x)$. Moreover, *u* satisfies the Euler-Lagrange equation

$$\begin{aligned} \int _{\Omega } (\nabla u \cdot \nabla \xi + V^{+} u\xi ){} = \int _{\Omega } \widehat{\xi } \,\mathrm {d}\nu {} \quad \text {for every }\xi \in W_{0}^{1, 2}(\Omega ) \cap L^{\infty }(\Omega ). \end{aligned}$$

Given a sequence of radial mollifiers $(\rho _{n})_{n \in {\mathbb N}}$ in $C_{c}^{\infty }(B_{1})$, let $u_{n}$ be the duality solution of (3.2) with datum $\rho _{n} * \nu$, so that by uniqueness $u_{n} = \zeta _{\rho _{n} * \nu }$ . By (3.5), for every $n \in {\mathbb N}$ we then have

3.9 $$\begin{aligned} \widehat{u_{n}} = 0 \quad \text {in }S. \end{aligned}$$

We claim that

3.10 $$\begin{aligned} u_{n} \rightarrow u \quad \text {in }W_{0}^{1, 2}(\Omega ). \end{aligned}$$

To this end, we subtract the Euler-Lagrange equations satisfied by $u_{n}$ and *u*, and apply $u_{n} - u$ as test function. Using the nonnegativity of $V^{+}$, we get

3.11 $$\begin{aligned} \int _{\Omega }{|\nabla (u_{n} - u)|^{2}} \le \int _{\Omega }{(u_{n} - u) \rho _{n} * \nu } - \int _{\Omega }{\widehat{u_{n} - u} \,\mathrm {d}\nu }. \end{aligned}$$

Since the sequence $(u_{n})_{n \in {\mathbb N}}$ is bounded in $W_{0}^{1, 2}(\Omega )$ and converges to *u* in $L^{1}(\Omega )$, we have weak convergence in $W_{0}^{1, 2}(\Omega )$. From (3.8) with $U = \Omega$, we then get

$$\begin{aligned} \lim _{n \rightarrow \infty }{\int _{\Omega }{\widehat{u_{n} - u} \,\mathrm {d}\nu }} = 0. \end{aligned}$$

For the first integral in the right-hand side of (3.11), we apply Fubini’s theorem,

3.12 $$\begin{aligned} \int _{\Omega }{(u_{n} - u) \rho _{n} * \nu } = \int _{\Omega }{{\rho _{n}} * (u_{n} - u) \,\mathrm {d}\nu }. \end{aligned}$$

Extending $u_{n} - u$ by 0 in ${\mathbb R}^{N} \setminus \Omega$ , we have that $u_{n} - u \in W^{1, 2}({\mathbb R}^{N})$. The sequence $({\rho _{n}} * (u_{n} - u))_{n \in {\mathbb N}}$ is supported in a smooth bounded open set $U \Supset \Omega$ and converges weakly to zero in $W^{1, 2}_{0}(U)$. From (3.8) and (3.12), we get

$$\begin{aligned} \lim _{n \rightarrow \infty }{\int _{\Omega }{(u_{n} - u) \rho _{n} * \nu }} = 0. \end{aligned}$$

Thus, as $n \rightarrow \infty$ in (3.11),

$$\begin{aligned} \lim _{n \rightarrow \infty }{\int _{\Omega }{|\nabla (u_{n} - u)|^{2}}} = 0, \end{aligned}$$

which implies (3.10). Now passing to a subsequence $(u_{n_{j}})_{j \in {\mathbb N}}$ , one deduces that

$$\begin{aligned} \widehat{u_{n_{j}}} \rightarrow \widehat{u} \quad \text {quasi-everywhere in } \Omega . \end{aligned}$$

In view of (3.9), the conclusion holds when $F * |\nu |$ is bounded.

In the case of a general measure $\nu \in \mathcal {M}(\Omega )$, it suffices to prove that the truncated function $T_{1}(u)$ satisfies the conclusion. Observe that $T_{1}(u) \in W_{0}^{1, 2}(\Omega ) \cap L^{2}(\Omega ; V^{+} \,\mathrm {d}x)$ and

$$\begin{aligned} - \Delta T_{1}(u) + V^{+} T_{1}(u) = \widetilde{\nu } \quad \text {in the sense of distributions in }\Omega , \end{aligned}$$

for some diffuse measure $\widetilde{\nu }\in \mathcal {M}(\Omega )$ ; see [3, 4, 7]. By a classical property in Potential Theory [12, Theorem 3.6.3] applied to $\widetilde{\nu }^{+}$ and $\widetilde{\nu }^{-}$, this measure $\widetilde{\nu }$ can be strongly approximated in $\mathcal {M}(\Omega )$ by a sequence of measures $(\nu _{j})_{j \in {\mathbb N}}$ such that $|\nu _{j}|$ has bounded Newtonian potential, for which the conclusion holds from the first part of the proof.

Denote by $v_{n}$ the duality solution of (3.2) with datum $\nu _{n}$. By uniqueness of duality solutions, $v_{n}$ and $T_{1}(u)$ are also minimizers of their associated energy functionals and therefore satisfy an Euler-Lagrange equation. Subtracting those equations, we then have

$$\begin{aligned} \int _{\Omega } \nabla (v_{n} - T_{1}(u)) \cdot \nabla \xi + V^{+}(v_{n} - T_{1}(u))\xi {} = \int _{\Omega } {\widehat{\xi }} \,\mathrm {d}(\nu _{n} - \widetilde{\nu }), \end{aligned}$$

for every $\xi \in W_{0}^{1, 2}(\Omega ) \cap L^{2}(\Omega ; V^{+} \,\mathrm {d}x)$. We now fix $k > 0$. Applying the identity above with test function $\xi = T_{k}(v_{n} - T_{1}(u))$, by nonnegativity of $V^{+}$ we get

$$\begin{aligned} \int _{\Omega } \bigl |\nabla \bigl (T_{k}(v_{n} - T_{1}(u))\bigr )\bigr |^{2} \le \int _{\Omega } \widehat{T_{k}(v_{n} - T_{1}(u))} \,\mathrm {d}(\nu _{n} - \widetilde{\nu }) \le k \int _{\Omega } \,\mathrm {d}|\nu _{n} - \widetilde{\nu }|. \end{aligned}$$

Thus, as $$n \rightarrow \infty $$,

$$\begin{aligned} T_{k}(v_{n} - T_{1}(u)) \rightarrow 0 \quad \text {in }W_{0}^{1, 2}(\Omega ). \end{aligned}$$

Since $\widehat{v_{n}} = 0$ quasi-everywhere in *S*, we conclude that

$$\begin{aligned} T_{1}(\widehat{u}) = \widehat{T_{1}(u)} = 0 \quad \text {quasi-everywhere in }S. \end{aligned}$$

$$\square $$

## Localization on $D_{i}$

In this section, we prove that duality solutions of (3.2) and functions in $W^{1,2}_{0}(\Omega ) \cap L^{2}(\Omega ; V^{+} \,\mathrm {d}x)$ can be localized on each component $D_{i}$ .

### Proposition 4.1

Let $i \in I$. If *u* is a duality solution of (3.2) with datum $\nu \in \mathcal {M}(\Omega )$, then $u\chi _{D_i}$ is a duality solution of (3.2) with datum $\nu \lfloor _{D_i}{}$.

We rely on an orthogonality property of the Green’s functions $G_{x}$ that is implicitly used in [19] to identify the components $D_{i}$ . It relies on (3.7) above and [19, Proposition 9.1], and is valid for each $i \in I$:

(i): For every $x \in \Omega \setminus D_{i}$ , we have $\widehat{G_{x}} = 0$ in $D_{i}$ ;
(ii): For every $x \in D_{i}$ , we have $\widehat{G_{x}} = 0$ in $\Omega \setminus D_{i}$ .

### Proof of Proposition 4.1

By the representation formula (3.6), the duality solution *v* of

4.1 $$\begin{aligned} \left\{ \begin{aligned}&-\Delta v + V^{+} v = \nu \lfloor _{D_{i}}&\quad \text {in } \Omega ,\\&v = 0&\quad \text {on } \partial \Omega , \end{aligned} \right. \end{aligned}$$

satisfies

4.2 $$\begin{aligned} v(x) = \int _{\Omega }\widehat{G_x} \,\mathrm {d}\nu \lfloor _{D_{i}}{} = \int _{D_{i}}\widehat{G_x} \,\mathrm {d}\nu \quad \text {for almost every }x \in \Omega . \end{aligned}$$

By (*i*),

4.3 $$\begin{aligned} \int _{D_{i}}\widehat{G_x} \,\mathrm {d}\nu = 0 \quad \text {for every }x \in \Omega \setminus D_{i} \end{aligned}$$

and, by (*ii*),

4.4 $$\begin{aligned} \int _{D_{i}}\widehat{G_x} \,\mathrm {d}\nu {} = \int _{\Omega }\widehat{G_x} \,\mathrm {d}\nu \quad \text {for every }x \in D_{i}\,. \end{aligned}$$

We conclude from (3.6) and (4.2), (4.3) and (4.4) that $v = u \chi _{D_{i}}$ almost everywhere in $\Omega$. Hence, $u\chi _{D_{i}}$ is the duality solution of (4.1). $\square$

As a consequence of Proposition 4.1, we deduce a localized strong maximum principle for duality solutions of (3.2). We first recall that the strong maximum principle holds for $\zeta _{g}$ with nonnegative $g \in L^{\infty }(\Omega )$ in the sense that, for each $i \in I$, we have that either $\widehat{\zeta _{g}} > 0$ on $D_{i}$ or $\widehat{\zeta _{g}} \equiv 0$ on $D_{i}$ ; see [19, Theorem 12.1]. Next, every duality solution *u* of (3.2) with nonnegative data $\nu$ can be bounded from below by some duality solution with nonnegative datum in $L^{\infty }(\Omega )$. More precisely, by [19, Proposition 7.1], there exists a bounded nondecreasing continuous function $Q : {\mathbb R}_{+} \rightarrow {\mathbb R}_{+}$ with $Q(t) > 0$ for $t > 0$ such that

4.5 $$\begin{aligned} u \ge \zeta _{Q(u)} \quad \text {almost everywhere in } \Omega . \end{aligned}$$

A valid choice of function *Q* satisfying (4.5) is $Q(t) = \frac{\alpha -1}{C\alpha }\min {\{t^\alpha ,1\}}$, where $\alpha >1$ and $C > 0$ is a constant that depends on $\alpha$ and $\Omega$.

### Corollary 4.2

Let $i \in I$ and let *u* be a nonnegative duality solution of (3.2) with datum $\nu \in \mathcal {M}(\Omega )$. If $\nu \ge 0$ in $D_{i}$ and $\int _{D_{i}} u > 0$, then

### Proof

Let $\bar{u}_{i} = u \chi _{D_{i}}$ . Then, $\bar{u}_{i}$ is a duality solution of (3.2) with nonnegative datum $\nu \lfloor _{D_{i}}$ . By the comparison principle (4.5),

$$\begin{aligned} u \ge \bar{u}_{i} \ge \zeta _{Q(\bar{u}_{i})} \quad \text {almost everywhere in }\Omega . \end{aligned}$$

Since $\int _{D_{i}} u > 0$, the function $Q(\bar{u}_{i})$ is nonzero on $D_{i}$ . Then, by the strong maximum principle, we have $\widehat{\zeta _{Q(\bar{u}_{i})}} > 0$ on $D_{i}$ . Thus, for every $x \in D_{i}$ ,$\square$

We now prove that decomposition (1.4) of $\Omega \setminus S$ in terms of its components $D_{i}$ induces a natural splitting (1.5) of functions in $W_{0}^{1, 2}(\Omega ) \cap L^{2}(\Omega ; V^{+} \,\mathrm {d}x)$.

### Proposition 4.3

If $\xi \in W_{0}^{1, 2}(\Omega ) \cap L^{2}(\Omega ; V^{+} \,\mathrm {d}x)$, then, for every $i \in I$, we have $\xi \chi _{D_{i}} \in W_{0}^{1, 2}(\Omega )$ and

$$\begin{aligned} \xi = \sum _{i \in I}{\xi \chi _{D_{i}}} \quad \text {almost everywhere in }\Omega . \end{aligned}$$

We begin with the following

### Lemma 4.4

For every $\xi \in W_{0}^{1, 2}(\Omega ) \cap L^{2}(\Omega ; V^{+} \,\mathrm {d}x)$, we have $\xi = 0$ almost everywhere in *S*.

### Proof of Lemma 4.4

We first observe that

4.6 $$\begin{aligned} \zeta _{\chi _{S}} = 0 \quad \text {almost everywhere in } \Omega . \end{aligned}$$

Indeed, since $\zeta _{1} = 0$ almost everywhere in *S*, by (3.4) we have

$$\begin{aligned} \int _{\Omega } \zeta _{\chi _{S}} = \int _{\Omega } \zeta _{1}\, \chi _{S} = 0. \end{aligned}$$

Since $\zeta _{\chi _{S}}$ is nonnegative, (4.6) follows.

Given $\xi \in W_{0}^{1, 2}(\Omega ) \cap L^{2}(\Omega ; V^{+} \,\mathrm {d}x)$, let us assume by contradiction that $S \cap \{\xi \ne 0\}$ has positive Lebesgue measure. Hence, $\int _{\Omega } |\xi | \, \chi _{S} > 0$. Computing the functional

$$\begin{aligned} \eta \in W_{0}^{1, 2}(\Omega ) \cap L^{2}(\Omega ; V^{+} \,\mathrm {d}x) \longmapsto \frac{1}{2} \int _{\Omega } (|\nabla \eta |^{2} + V^{+}\eta ^{2}) - \int _{\Omega } \eta \, \chi _{S} \end{aligned}$$

on $t |\xi |$ for $t > 0$ small, one concludes that its infimum is negative. Thus, the minimizer $\zeta _{\chi _{S}}$ is nontrivial, in contradiction with (4.6). Therefore, the set $S \cap \{\xi \ne 0\}$ has Lebesgue measure zero. $\square$

Lemma 4.4 suffices for our purposes and is trivially satisfied when *S* is negligible for the Lebesgue measure. A small adaptation of the proof yields a sharper version in terms of the $W^{1, 2}$ capacity:

### Proposition 4.5

For every $\xi \in W_{0}^{1, 2}(\Omega ) \cap L^{2}(\Omega ; V^{+} \,\mathrm {d}x)$, we have $\widehat{\xi } = 0$ quasi-everywhere in *S*.

### Proof of Proposition 4.5

We assume by contradiction that $S \cap \{\widehat{\xi } \ne 0\}$ has positive $W^{1, 2}$ capacity. Then, there exist a compact set $K \subset S \cap \{\widehat{\xi } \ne 0\}$ with positive capacity and a nonnegative diffuse measure $\nu \in \mathcal {M}(\Omega ) \cap (W_{0}^{1, 2}(\Omega ))'$ supported in *K* with $\nu (K) > 0$ ; see [23, Proposition A.17]. We then have that $\int _{\Omega } \widehat{\xi } \,\mathrm {d}\nu > 0$ and the functional

$$\begin{aligned} \eta \in W_{0}^{1, 2}(\Omega ) \cap L^{2}(\Omega ; V^{+} \,\mathrm {d}x) \longmapsto \frac{1}{2} \int _{\Omega } (|\nabla \eta |^{2} + V^{+}\eta ^{2}) - \int _{\Omega } \widehat{\eta } \, \,\mathrm {d}\nu \end{aligned}$$

has a nontrivial nonnegative minimizer *v*, which is the duality solution of (3.2). In particular, since $\nu$ is supported in *S*,

$$\begin{aligned} \int _{\Omega } v = \int _{\Omega } \widehat{\zeta _{1}} \,\mathrm {d}\nu = 0. \end{aligned}$$

We thus have a contradiction, whence $S \cap \{\widehat{\xi } \ne 0\}$ has $W^{1, 2}$ capacity zero. $\square$

### Proof of Proposition 4.3

Since

$$\begin{aligned} \xi = \sum _{i \in I}{\xi \chi _{D_{i}}} + \xi \chi _{S} \end{aligned}$$

and, by Lemma 4.4, $\xi = 0$ almost everywhere in *S*, we are left to prove that $\xi \chi _{D_{i}} \in W_{0}^{1, 2}(\Omega )$ for every $i \in I$.

Given $\Phi \in C^{\infty }(\overline{\Omega }; {\mathbb R}^{N})$, let *v* be the duality solution of (3.2) with datum $\nu = {{\,\mathrm{div}\,}}{\Phi } \,\mathrm {d}x$. Then, by Proposition 4.1,  is the duality solution of (3.2) with datum $\nu = ({{\,\mathrm{div}\,}}{\Phi })\chi _{D_{i}} \,\mathrm {d}x$. By uniqueness, *v* and $\bar{v}_{i}$ are also variational solutions for the same data. In particular, they both belong to $W_{0}^{1, 2}(\Omega ) \cap L^{2}(\Omega ; V^{+} \,\mathrm {d}x)$ and satisfy the associated Euler–Lagrange equations.

We now observe that

4.7 $$\begin{aligned} \nabla {\bar{v}}_{i} = (\nabla v) \chi _{D_{i}} \quad \text {almost everywhere in }\Omega . \end{aligned}$$

Indeed, this follows from the facts that $\nabla {\bar{v}}_{i} = 0$ almost everywhere in $\{{\bar{v}}_{i} = 0\} \supset \Omega \setminus D_{i}$ and $\nabla ({\bar{v}}_{i} - v) = 0$ almost everywhere in $\{{\bar{v}}_{i} - v = 0\} \supset D_{i}$. For $\xi \in W_{0}^{1, 2}(\Omega ) \cap L^{2}(\Omega ; V^{+} \,\mathrm {d}x)$, using the Euler–Lagrange equation satisfied by ${\bar{v}}_{i}$ and (4.7) we then have

$$\begin{aligned} \int _{\Omega } \xi \chi _{D_{i}} {{\,\mathrm{div}\,}}{\Phi } = \int _{\Omega } \nabla \bar{v}_{i} \cdot \nabla \xi + V^{+} {\bar{v}}_{i} \xi {} = \int _{D_{i}} \nabla v \cdot \nabla \xi + V^{+} v \xi . \end{aligned}$$

Therefore,

4.8 $$\begin{aligned} \biggl | \int _{\Omega } \xi \chi _{D_{i}} {{\,\mathrm{div}\,}}{\Phi } \biggr |{} \le \Vert v\Vert \Vert \xi \Vert , \end{aligned}$$

where we use the normApplying *v* as a test function in the Euler–Lagrange equation satisfied by *v* itself, we have

$$\begin{aligned} \Vert v\Vert ^{2} = \int _{\Omega } v {{\,\mathrm{div}\,}}{\Phi } = - \int _{\Omega } \nabla v \cdot \Phi {} \le \Vert v\Vert \Vert \Phi \Vert _{L^{2}(\Omega )}. \end{aligned}$$

Thus, $\Vert v\Vert \le \Vert \Phi \Vert _{L^{2}(\Omega )}$. Inserting this estimate in (4.8) we get

4.9 $$\begin{aligned} \biggl | \int _{\Omega } \xi \chi _{D_{i}} {{\,\mathrm{div}\,}}{\Phi } \biggr |{} \le \Vert \Phi \Vert _{L^{2}(\Omega )} \Vert \xi \Vert {} \quad \text {for every }\Phi \in C^{\infty }(\overline{\Omega }; {\mathbb R}^{N}). \end{aligned}$$

We deduce from the Riesz Representation Theorem that $\nabla (\xi \chi _{D_{i}}) \in L^{2}(\Omega ; {\mathbb R}^{N})$. Since $\Omega$ is smooth and (4.9) holds for every smooth $\Phi$ that need not have compact support in $\Omega$, we conclude that $\xi \chi _{D_{i}} \in W_{0}^{1, 2}(\Omega )$. $\square$

## Duality solutions for signed potentials

We begin by extending the definition of duality solutions to the Dirichlet problem

5.1 $$\begin{aligned} \left\{ \begin{aligned}&-\Delta u+ V u = \mu&\quad \text {in } \Omega ,\\&u = 0&\quad \text {on } \partial \Omega , \end{aligned} \right. \end{aligned}$$

with a signed potential *V*:

### Definition 5.1

Given $\mu \in \mathcal {M}(\Omega )$, we say that $u \in L^{1}(\Omega )$ is a *duality solution* of (5.1) whenever $V^{-}u \in L^{1}(\Omega )$ and

$$\begin{aligned} \int _{\Omega }u f = \int _{\Omega } \widehat{\zeta _f} \,\mathrm {d}\mu + \int _{\Omega } \zeta _f V^{-}u \quad \text {for every } f \in L^{\infty }(\Omega ), \end{aligned}$$

where $\zeta _{f}$ is the minimizer of (3.1).

It is convenient to observe that, as the test functions $\zeta _{f}$ depend on $V^{+}$ and $\widehat{\zeta _{f}} = \zeta _{f}$ almost everywhere in $\Omega$, a duality solution of (5.1) is also a duality solution of

5.2 $$\begin{aligned} \left\{ \begin{aligned}&-\Delta u+ V^{+} u = \mu + V^{-} u&\quad \text {in } \Omega ,\\&u = 0&\quad \text {on } \partial \Omega , \end{aligned} \right. \end{aligned}$$

where $\mu + V^{-} u$ is regarded as the datum of the problem. In particular, the precise representative $\widehat{u}$ is also well-defined quasi-everywhere in $\Omega$ and we have the following direct consequences of Propositions 3.3 and 4.1, respectively:

### Corollary 5.2

If *u* is a duality solution of (5.1) with datum $\mu \in \mathcal {M}(\Omega )$, then $\widehat{u} = 0$ quasi-everywhere in *S*.

### Corollary 5.3

Let $i \in I$. If *u* is a duality solution of (5.1) with datum $\mu \in \mathcal {M}(\Omega )$, then $u\chi _{D_i}$ is a duality solution of (5.1) with datum $\mu \lfloor _{D_i}{}$ .

We now rely upon [19, Proposition 4.1] for the operator $-\Delta + V^{+}$ to deduce that a duality solution is a distributional solution with possibly different datum:

### Proposition 5.4

If *u* is a duality solution of (5.1) with datum $\mu \in \mathcal {M}(\Omega )$, then $u \in W_{0}^{1, 1}(\Omega )$ and

$$\begin{aligned} - \Delta u + Vu = \mu \lfloor _{\Omega \setminus S}{} - \tau {} \quad \text {in the sense of distributions in }\Omega , \end{aligned}$$

where $\tau \in \mathcal {M}(\Omega )$ is a diffuse measure such that $|\tau |(\Omega \setminus S) = 0$.

Since $\tau$ is diffuse and carried by *S*, under the assumption

5.3 $$\begin{aligned} {{\,\mathrm{cap}\,}}_{W^{1, 2}}{(S)} = 0, \end{aligned}$$

it then follows that $\tau = 0$. The smallness condition (5.3) is directly verified with the existence of $\xi \in W_{0}^{1, 2}(\Omega ) \cap L^{2}(\Omega ; V^{+} \,\mathrm {d}x)$ whose level set $\{\widehat{\xi } = 0\}$ has $W^{1, 2}$ capacity zero; see Proposition 4.5.

### Proof (Proof of Proposition 5.4)

Since *u* is a duality solution of (5.2) involving a finite measure in $\Omega$, we have $u \in W_{0}^{1, 1}(\Omega )$. Take the duality solutions $u_{1}$ and $u_{2}$ of (3.2) with data $(\mu + V^{-}u \,\mathrm {d}x)^{+}$ and $(\mu + V^{-}u \,\mathrm {d}x)^{-}$, respectively. By [19, Proposition 4.1], there exist nonnegative diffuse measures $\tau _{1}$ and $\tau _{2}$ such that $\tau _{1}(\Omega \setminus S) = \tau _{2}(\Omega \setminus S) = 0$ with

$$\begin{aligned} - \Delta u_{1} + V^{+}u_{1} = (\mu + V^{-}u \,\mathrm {d}x)^{+}\lfloor _{\Omega \setminus S}{} - \tau _{1}{} \end{aligned}$$

and

$$\begin{aligned} - \Delta u_{2} + V^{+}u_{2} = (\mu + V^{-}u \,\mathrm {d}x)^{-}\lfloor _{\Omega \setminus S}{} - \tau _{2}{} \end{aligned}$$

in the sense of distributions in $\Omega$. From Proposition 3.2, one has $u = 0$ almost everywhere in *S*. Thus,

5.4 $$\begin{aligned} V^{-}u\chi _{\Omega \setminus S} = V^{-}u \quad \text {almost everywhere in }\Omega . \end{aligned}$$

Subtracting the equations satisfied by $u_{1}$ and $u_{2}$, and using (5.4), we then get

$$\begin{aligned} - \Delta u + V^{+}u = \mu \lfloor _{\Omega \setminus S}{} + V^{-}u - \tau {} \quad \text {in the sense of distributions in }\Omega , \end{aligned}$$

where . $$\square $$

Even for a nonnegative potential *V* the measure $\tau$ which appears in Proposition 5.4 can be nonzero:

### Example 5.5

Given $\alpha \ge 1$, let $V : B_{1} \rightarrow [0, +\infty ]$ be defined for $x = (x_{1}, \ldots , x_{N})$ by

$$\begin{aligned} V(x) = \frac{1}{|x_{1}|^{\alpha }}. \end{aligned}$$

In this case, $S = B_{1} \cap \{x_{1} = 0\}$ and then $B_{1} \setminus S$ has two connected components. Since $\zeta _{1}$ is a duality solution with constant datum 1, we have

$$\begin{aligned} - \Delta \zeta _{1} + V \zeta _{1} = 1 - \tau \quad \text {in the sense of distributions in }B_{1}\,, \end{aligned}$$

for some finite measure $\tau$ supported in $\{x_{1} = 0\}$. When $\alpha \ge 2$, we have $\tau = 0$, but when $1 \le \alpha < 2$ the Hopf lemma holds in each component of $B_{1} \setminus S$ and implies that $\tau (S) > 0$ ; see [17, Theorem 9.1].

### Corollary 5.6

If *u* is a nonnegative distributional solution of (5.1) with datum $\mu \in \mathcal {M}(\Omega )$, then $\mu \lfloor _{S}{} \le 0$.

### Proof

We recall that a distributional solution is also a duality solution for the same datum. By comparison with the conclusion of Proposition 5.4, we deduce that $\mu \lfloor _{S}{} = -\tau$ in the sense of distributions in $\Omega$, and then equality holds as measures; see [23, Proposition 6.12]. We are left to show that $\tau \ge 0$. To this end, we first identify the diffuse part $(\Delta u)_{\mathrm {d}}$ of the measure $\Delta u$ with respect to the $W^{1, 2}$ capacity. Since $Vu = 0$ almost everywhere in *S* and $\tau = -\mu \lfloor _{S}{}$ is diffuse, we have

5.5 $$\begin{aligned} (\Delta u)_{\mathrm {d}} = \tau {} \quad \text {in } S. \end{aligned}$$

On the other hand, as a consequence of Kato’s inequality, by nonnegativity of *u* in $\Omega$ we have

5.6 $$\begin{aligned} (\Delta u)_{\mathrm {d}} \ge 0 \quad \text {in } \{\widehat{u} = 0\}\,; \end{aligned}$$

see [23, Eq. (6.5)]. By Corollary 5.2, we have $\widehat{u} = 0$ quasi-everywhere in *S*. It thus follows from (5.5) and (5.6) that $\mu \lfloor _{S}{} = - \tau \le 0$. $\square$

## Approximation scheme

Existence of a unique duality solution of (3.2) entitles us to construct a sequence of duality solutions for a family of truncated problems associated to (5.1) with signed *V*.

### Proposition 6.1

Let $\mu$ be a nonnegative measurable function on $\Omega$ and let $(u_{n})_{n \in {\mathbb N}}$ be the sequence defined by induction as $u_{0} = 0$ and, for $n \ge 1$, $u_{n}$ is the duality solution of

$$\begin{aligned} \left\{ \begin{aligned} -\Delta u_{n} + V^+ u_{n}&= T_{n}(\mu ) + T_{n}(V^-) u_{n-1}&\quad \text {in } \Omega ,\\ u_{n}&= 0&\quad \text {on } \partial \Omega . \end{aligned} \right. \end{aligned}$$

Then, for every $n \in {\mathbb N}$, we have $u_{n} \in W_{0}^{1, 2}(\Omega ) \cap L^{2}(\Omega ; V^{+} \,\mathrm {d}x) \cap L^{\infty }(\Omega )$ and

$$\begin{aligned} 0\le u_{n}\le u_{n+1} \quad \hbox { almost everywhere in}\ \Omega . \end{aligned}$$

### Proof

For every nonnegative $f \in L^{\infty }(\Omega )$, we have $\zeta _{f} \ge 0$ almost everywhere in $\Omega$. Thus, by nonnegativity of $\mu$,

$$\begin{aligned} \int _{\Omega } u_{1}f = \int _{\Omega } T_{1}(\mu ) \zeta _{f} \ge 0. \end{aligned}$$

Hence, $u_{1} \ge 0 = u_{0}$ almost everywhere in $\Omega$. We next assume that the conclusion holds for some $n \in {\mathbb N}$. By nonnegativity of $\mu$, $T_{n+2}(\mu ) \ge T_{n+1}(\mu )$. Then, for every nonnegative $f \in L^{\infty }(\Omega )$,

$$\begin{aligned} \int _{\Omega } (u_{n + 2} - u_{n + 1}) f \ge \int _{\Omega } (T_{n + 2}(V^{-})u_{n + 1} - T_{n + 1}(V^{-})u_{n}) \zeta _{f}. \end{aligned}$$

By the induction assumption, the integrand in the right-hand side is nonnegative and we deduce that $u_{n+2} - u_{n+1} \ge 0$ almost everywhere in $\Omega$. Finally, since $T_{n}(\mu ) \in L^{\infty }(\Omega )$, we have by induction that $u_{n} \in L^{\infty }(\Omega )$ for every $n \in {\mathbb N}$. As $u_{n} \in L^1(\Omega ; V^+\,\mathrm {d}x)$, we then have $u_{n} \in L^2(\Omega ; V^+\,\mathrm {d}x)$. Finally, since $\Delta u_{n}$ is a finite measure in $\Omega$, we also have by interpolation that $u_{n} \in W_{0}^{1,2}(\Omega )$. $\square$

## Variational setting

Throughout the section, we assume that there exists a measurable function $w_{i} : D_{i} \rightarrow (0, +\infty )$ such that (1.8) holds, whence

$$\begin{aligned} \mathcal {H}_{i}(\Omega ) \subset L^{2}(D_{i}; w_{i} \,\mathrm {d}x). \end{aligned}$$

Denoting by *E* the functional defined in $\mathcal {H}_{i}(\Omega )$ by (1.9), the following property is standard in the Calculus of Variations:

### Proposition 7.1

For every $h\in L^2(D_{i}; w_{i} \,\mathrm {d}x)$, the functional *E* has a unique minimizer $\theta _{i, h}$ and any minimizing sequence of *E* is a Cauchy sequence in $\mathcal {H}_{i}(\Omega )$.

### Proof

Uniqueness of the minimizer follows from the parallelogram identity satisfied by the norm $\Vert \cdot \Vert _{i}$ which yields

7.1 $$\begin{aligned} \frac{1}{2} \Vert u - v\Vert _{i}^{2} = E(u) + E(v) - 2 E\Bigl ( \frac{u + v}{2} \Bigr ){} \quad \text {for every }u, v \in \mathcal {H}_{i}(\Omega ). \end{aligned}$$

If *u* and *v* are both minimizers of *E*, then $\Vert u - v\Vert _{i}^{2} \le 0$.

We now observe that *E* is bounded from below, whence $\inf {E} \in {\mathbb R}$. Taking a minimizing sequence $(\xi _j)_{j\in \mathbb {N}}$ , it follows from (7.1) that, for every $j, k \in {\mathbb N}$,

$$\begin{aligned} \frac{1}{2} \Vert \xi _{j} - \xi _{k}\Vert _{i}^{2} = E(\xi _{j}) + E(\xi _{k}) - 2 E\Bigl ( \frac{\xi _{j} + \xi _{k}}{2} \Bigr ){} \le E(\xi _{j}) + E(\xi _{k}) - 2 \inf {E}. \end{aligned}$$

Observe that the right-hand side converges to zero as $j, k \rightarrow \infty$. Hence, $(\xi _j)_{j\in \mathbb {N}}$ is a Cauchy sequence and converges in $\mathcal {H}_{i}(\Omega )$ to the unique minimizer of *E*. $\square$

### Proposition 7.2

If $h\in L^2(D_{i}; w_{i} \,\mathrm {d}x)$ is nonnegative, then $\theta _{i, h} \ge 0$ almost everywhere in $D_{i}$ .

### Proof

Let $(\xi _{j})_{j \in {\mathbb N}}$ be a minimizing sequence in $H_{i}(\Omega )$ of the functional *E*. For each $j \in {\mathbb N}$, we write

$$\begin{aligned} E(\xi _{j}) = E(\xi _{j}^{+}) + E(-\xi _{j}^{-}). \end{aligned}$$

Since *h* is nonnegative and (1.8) holds, we have $E(-\xi _{j}^{-}) \ge 0$ and then

$$\begin{aligned}{} E(\xi _{j}) \ge E(\xi _{j}^{+}) \end{aligned}$$

Thus, $(\xi _{j}^{+})_{j \in {\mathbb N}}$ is also a minimizing sequence. By Proposition 7.1, it converges to $\theta _{i, h}$ in $\mathcal {H}_{i}(\Omega )$ and then also in $L^{2}(D_{i}; w_{i} \,\mathrm {d}x)$. In particular, $\theta _{i, h} \ge 0$ almost everywhere in $D_{i}$ . $\square$

As an alternative to the previous proof based on minimizing sequences, one may rely on the decomposition $\theta _{i, h} = \theta _{i, h}^{+} - \theta _{i, h}^{-}$ in $\mathcal {H}_{i}(\Omega )$ as a difference between positive and negative parts. It then suffices to use $\theta _{i, h}^{-}$ in the Euler–Lagrange equation to deduce in a standard way that $\theta _{i, h}^{-} = 0$. The possibility of such an approach is ensured by the following

### Proposition 7.3

Let $i \in I$. If $u, v \in \mathcal {H}_{i}(\Omega )$, then there exist $f, g \in \mathcal {H}_{i}(\Omega )$ such that $f = \min {\{u, v\}}$ and $g = \max {\{u, v\}}$ almost everywhere in $D_{i}$ and

$$\begin{aligned}{} \Vert f\Vert _{i}^{2} + \Vert g\Vert _{i}^{2} \le \Vert u\Vert _{i}^{2} + \Vert v\Vert _{i}^{2}. \end{aligned}$$

We rely on the following closure property which is a convenient tool to identify the weak limit of a sequence in $\mathcal {H}_{i}(\Omega )$ :

### Lemma 7.4

Let $(v_{n})_{n \in {\mathbb N}}$ be a sequence in $\mathcal {H}_{i}(\Omega )$. If $v_{n} \rightarrow v$ almost everywhere in $D_{i}$ and $v_{n} \rightharpoonup \widetilde{v}$ in $\mathcal {H}_{i}(\Omega )$, then $v = \widetilde{v}$ almost everywhere in $D_{i}$ .

### Proof of Lemma 7.4

By the Banach–Saks property in a Hilbert space (which is a more comfortable and precise version of Mazur’s lemma), we can take a subsequence $(v_{n_{j}})_{j \in {\mathbb N}}$ that converges strongly to $\widetilde{v}$ in $\mathcal {H}_{i}(\Omega )$ in the Cesàro sense, that is

$$\begin{aligned}{} \frac{1}{N + 1}\sum _{j = 0}^{N}{v_{n_{j}}} \rightarrow \widetilde{v} \quad \text {in }\mathcal {H}_{i}(\Omega ), \end{aligned}$$

and then also in $L^{2}(D_{i}; w_{i} \,\mathrm {d}x)$. By pointwise convergence of $(v_{n_{j}})_{j \in {\mathbb N}}$ to *v* in $D_{i}$ , we deduce that $v = \widetilde{v}$ almost everywhere in $D_{i}$ . $\square$

### Proof of Proposition 7.3

Take sequences $(u_{n})_{n \in {\mathbb N}}$ and $(v_{n})_{n \in {\mathbb N}}$ in $H_{i}(\Omega )$ such that

$$\begin{aligned}{} u_{n} \rightarrow u, \quad v_{n} \rightarrow v \quad {} \text {in } \mathcal {H}_{i}(\Omega )\hbox { and almost everywhere in }D_{i}\,. \end{aligned}$$

For every $n \in {\mathbb N}$, we have

$$\begin{aligned}{} (\min {\{u_{n}, v_{n}\}})^{2} + (\max {\{u_{n}, v_{n}\}})^{2} = u_{n}^{2} + v_{n}^{2} \end{aligned}$$

and

$$\begin{aligned}{} |\nabla \min {\{u_{n}, v_{n}\}}|^{2} + |\nabla \max {\{u_{n}, v_{n}\}}|^{2} = |\nabla u_{n}|^{2} + |\nabla v_{n}|^{2} \end{aligned}$$

almost everywhere in $D_{i}$. Thus,

$$\begin{aligned}{} \Vert \min {\{u_{n}, v_{n}\}}\Vert _{i}^{2} + \Vert \max {\{u_{n}, v_{n}\}}\Vert _{i}^{2} = \Vert u_{n}\Vert _{i}^{2} + \Vert v_{n}\Vert _{i}^{2}. \end{aligned}$$

In particular, the sequences $(\min {\{u_{n}, v_{n}\}})_{n \in {\mathbb N}}$ and $(\max {\{u_{n}, v_{n}\}})_{n \in {\mathbb N}}$ are bounded in $\mathcal {H}_{i}(\Omega )$. Hence, we may extract subsequences that converge weakly in $\mathcal {H}_{i}(\Omega )$ to *f* and *g*, respectively. As they converge almost everywhere to $\min {\{u, v\}}$ and $\max {\{u, v\}}$ in $D_{i}$ , the conclusion follows from Lemma 7.4 and the lower semicontinuity of the norm under weak convergence. $\square$

In contrast with the existence of a positive and negative parts in $\mathcal {H}_{i}(\Omega )$, it is unclear whether every $u \in \mathcal {H}_{i}(\Omega )$ admits a truncation $T_{k}(u) \in \mathcal {H}_{i}(\Omega )$ for every $k > 0$. Let us now show that the minimizer $\theta _{i,h}$ is an upper bound for the approximating sequence constructed in Sect. 6.

### Proposition 7.5

If $h \in L^{2}(D_{i}; w_{i} \,\mathrm {d}x)$ is nonnegative and if $(u_{n})_{n \in {\mathbb N}}$ is the sequence defined in Proposition 6.1 with $\mu = w_{i}h \chi _{D_{i}}$, then, for every $n \in {\mathbb N}$, we have

$$\begin{aligned} 0 \le u_{n} \le \theta _{i, h} \quad \text {almost everywhere in } D_{i}\,. \end{aligned}$$

### Proof

Since $u_{0} = 0$, the case $n = 0$ is a consequence of Proposition 7.2. We now assume by induction that the inequality holds for $n - 1$ for some $n \ge 1$. Our goal is to show that if $(\xi _{j})_{j \in {\mathbb N}}$ is a minimizing sequence in $H_{i}(\Omega )$ of the functional *E*, then $(\max {\{u_{n}, \xi _{j}\}})_{j \in {\mathbb N}}$ is also a minimizing sequence. To this end, we begin by observing that $u_{n} \in W^{1,2}_0(\Omega ) \cap L^2(\Omega ; V^+ \,\mathrm {d}x)$ is the minimizer of the functional $F_{n}$ defined on $W_{0}^{1, 2}(\Omega ) \cap L^{2}(\Omega ; V^{+} \,\mathrm {d}x)$ by

$$\begin{aligned} F_{n} (\eta ) = \frac{1}{2} \int _{\Omega }(|\nabla \eta |^{2} + V^{+}\eta ^{2}) - \int _{\Omega } \bigl (T_{n}(w_{i}h\chi _{D_{i}}) + T_{n}(V^{-}) u_{n - 1}\bigr ) \eta . \end{aligned}$$

In particular, for every $\xi \in H_{i}(\Omega )$,

7.2 $$\begin{aligned} F_{n}(u_{n}) \le F_{n}(\min {\{u_{n}, \xi \}}). \end{aligned}$$

We next show the following consequence of (7.2) for the functional *E* :

7.3 $$\begin{aligned} E(u_{n}) \le E(\min {\{u_{n}, \xi \}}) + \int _{\Omega } V^{-} (u_{n - 1} - \xi )^{+} (u_{n} - \xi )^{+}. \end{aligned}$$

Indeed, for every $\eta \in H_{i}(\Omega )$,

$$\begin{aligned}{} E(\eta ){} = F_{n}(\eta ){} - \frac{1}{2} \int _{\Omega } V^{-}\eta ^{2} + \int _{\Omega } T_{n}(V^{-}) u_{n-1} \eta - \int _{D_{i}} (w_{i} h - T_{n}(w_{i} h)) \eta . \end{aligned}$$

Applying (7.2) and $u_{n} \ge \min {\{u_{n}, \xi \}}$, we get

$$\begin{aligned}{} E(u_{n}) - E(\min {\{u_{n}, \xi \}})&\le - \frac{1}{2} \int _{\Omega } V^{-} (u_{n}^{2} - \min {\{u_{n}, \xi \}}^{2}) \\&\quad + \int _{\Omega } V^{-} u_{n-1} (u_{n} - \min {\{u_{n}, \xi \}})\\&=\quad \int _{\Omega } V^{-} \Bigl (u_{n - 1} - \frac{u_{n} + \min {\{u_{n}, \xi \}}}{2}\Bigr ) (u_{n} - \min {\{u_{n}, \xi \}}). \end{aligned}$$

Thus,

$$\begin{aligned}{} \begin{aligned} E(u_{n}) - E(\min {\{u_{n}, \xi \}})&\le \int _{\{u_{n} > \xi \}} V^{-} \Bigl (u_{n - 1} - \frac{u_{n} + \xi }{2}\Bigr )(u_{n} - \xi ) \\&\le \int _{\Omega } V^{-} (u_{n - 1} - \xi )^{+} (u_{n} - \xi )^{+}, \end{aligned} \end{aligned}$$

which gives (7.3).

Using a standard property of the maximum and minimum between two functions, we then get from (7.3) that

7.4 $$\begin{aligned} \begin{aligned} E(\max {\{u_{n}, \xi \}})&= E(\xi ) + E(u_{n}) - E(\min {\{u_{n}, \xi \}})\\&\le E(\xi ) + \int _{\Omega } V^{-} (u_{n - 1} - \xi )^{+} (u_{n} - \xi )^{+}. \end{aligned} \end{aligned}$$

As we mentioned above, we now apply this estimate to a minimizing sequence $(\xi _{j})_{j \in {\mathbb N}}$ in $H_{i}(\Omega )$ of the functional *E*. We first recall that, by Proposition 7.1, we have $\xi _{j} \rightarrow \theta _{i, h}$ in $\mathcal {H}_{i}(\Omega )$. Since the functions $u_{n}$ and $u_{n - 1}$ are bounded and belong to $L^{1}(\Omega ; V^{-} \,\mathrm {d}x)$, they also belong to $L^{2}(\Omega ; V^{-} \,\mathrm {d}x)$. By the Dominated Convergence Theorem and the induction assumption $u_{n - 1} \le \theta _{i, h}$ in $D_{i}$ , we then have

$$\begin{aligned}{} \lim _{j \rightarrow \infty }{\int _{\Omega } V^{-} (u_{n - 1} - \xi _{j})^{+} (u_{n} - \xi _{j})^{+}} = \int _{\Omega } V^{-} (u_{n - 1} - \theta _{i, h})^{+} (u_{n} - \theta _{i, h})^{+} = 0. \end{aligned}$$

We then deduce from (7.4) that

$$\begin{aligned}{} \limsup _{j \rightarrow \infty }{E(\max {\{u_{n}, \xi _{j}\}})} \le \lim _{j \rightarrow \infty }{E(\xi _{j})} + 0 = \inf {E}. \end{aligned}$$

Hence, $(\max {\{u_{n}, \xi _{j}\}})_{j \in {\mathbb N}}$ is also a minimizing sequence of *E* as claimed. Therefore, $\max {\{u_{n}, \xi _{j}\}} \rightarrow \theta _{i, h}$ in $\mathcal {H}_{i}(\Omega )$ as $j \rightarrow \infty$ and then, by (1.8),

$$\begin{aligned}{} u_{n} \le \max {\{u_{n}, \xi _{j}\}} \rightarrow \theta _{i, h} \quad \text {in } L^{2}(D_{i}; w_{i} \,\mathrm {d}x). \end{aligned}$$

Thus, $u_{n} \le \theta _{i, h}$ almost everywhere in $D_{i}$ . $\square$

We now identify the limit of the sequence $(u_{n})_{n \in {\mathbb N}}$ with the minimizer $\theta _{i, h}$ :

### Proposition 7.6

Let $h \in L^{2}(D_{i}; w_{i} \,\mathrm {d}x)$ be a nonnegative function and let $(u_{n})_{n \in {\mathbb N}}$ be the sequence defined in Proposition 6.1 with $\mu = w_{i}h \chi _{D_{i}}$. If $(u_{n})_{n \in {\mathbb N}}$ is bounded in $L^{2}(D_{i}; w_{i} \,\mathrm {d}x)$ and in $L^{1}(D_{i}; V^{-} \,\mathrm {d}x)$, then

$$\begin{aligned}{} \lim _{n \rightarrow \infty }{u_{n}} = \theta _{i, h} \quad \text {almost everywhere in }D_{i}\,. \end{aligned}$$

### Proof

We first show that $(u_{n})_{n \in {\mathbb N}}$ is bounded in $\mathcal {H}_{i}(\Omega )$. To this end, we recall that, for every $n \in {\mathbb N}$, we have $u_n\in H_i(\Omega )$ and

7.5 $$\begin{aligned} \int _{D_i} (\nabla u_n\cdot \nabla \xi + V^+ u_n \xi ){} = \int _{D_i} (T_{n}(w_{i} h) + T_{n}(V^{-}) u_{n-1})\xi \end{aligned}$$

for every $\xi \in W_{0}^{1, 2}(\Omega ) \cap L^{2}(\Omega ; V^{+} \,\mathrm {d}x).$ On the other hand, since $(u_{n})_{n \in {\mathbb N}}$ is nondecreasing,

$$\begin{aligned} \Vert u_{n}\Vert _{i}^{2} = \int _{D_{i}} (|\nabla u_{n}|^{2} + V u_{n}^{2}) \le \int _{D_{i}} (|\nabla u_{n}|^{2} + V^{+} u_{n}^{2} - T_{n}(V^{-}) u_{n - 1} u_{n}). \end{aligned}$$

Taking $\xi = u_{n}$ in (7.5), we get

$$\begin{aligned} \Vert u_{n}\Vert _{i}^{2} \le \int _{D_{i}} T_{n}(w_{i}h) u_{n} \le \int _{D_{i}} w_{i} h u_{n}. \end{aligned}$$

Since $h \in L^{2}(D_{i}; w_{i} \,\mathrm {d}x)$ and $(u_{n})_{n \in {\mathbb N}}$ is bounded in $L^{2}(D_{i}; w_{i} \,\mathrm {d}x)$, it follows from this estimate that $(u_{n})_{n \in {\mathbb N}}$ is also bounded in $\mathcal {H}_i(\Omega )$. Therefore, there exists a subsequence $(u_{n_{j}})_{j \in {\mathbb N}}$ such that

$$\begin{aligned} u_{n_{j}} \rightharpoonup \widetilde{u} \quad \text {in } \mathcal {H}_i(\Omega ). \end{aligned}$$

Since $(u_{n})_{n \in {\mathbb N}}$ is nondecreasing, it converges pointwise and then, by Lemma 7.4,

$$\begin{aligned} \widetilde{u} = \lim _{j \rightarrow \infty }{u_{n_{j}}} = \lim _{n \rightarrow \infty }{u_{n}} \quad \text {almost everywhere in }D_{i}\,. \end{aligned}$$

As $(u_{n})_{n \in {\mathbb N}}$ is bounded in $L^{1}(D_{i}; V^{-} \,\mathrm {d}x)$, we also have $\widetilde{u} \in L^{1}(D_{i}; V^{-} \,\mathrm {d}x)$.

Recalling that *E* has a unique minimizer $\theta _{i, h}$ in $\mathcal {H}_i(\Omega )$, to conclude the proof of the proposition it suffices to show that ${\widetilde{u}}$ satisfies the Euler–Lagrange equation:

7.6 $$\begin{aligned} ({\widetilde{u}}\vert \xi )_{i} = \int _{D_i} w_{i} h\xi \quad \text {for every }\xi \in \mathcal {H}_i(\Omega ), \end{aligned}$$

where $(\cdot \vert \cdot )_{i}$ denotes the inner product of $\mathcal {H}_i(\Omega )$ associated to its norm. Since we do not know whether ${\widetilde{u}} \in L^{2}(D_{i}; V \,\mathrm {d}x)$, one should be careful when letting $n \rightarrow \infty$ in (7.5).

To overcome this difficulty, we proceed with $\xi \in H_{i}(\Omega ) \cap L^{\infty }(\Omega )$. We rewrite (7.5) with $n = n_{j}$ as

7.7 $$\begin{aligned} (u_{n_{j}}\vert \xi )_{i} + \int _{D_{i}} (V^{-}u_{n_{j}} - T_{n_{j}}(V^{-}) u_{n_{j} - 1})\xi = \int _{D_{i}} T_{n_{j}}(w_{i}h) \xi . \end{aligned}$$

Since $0 \le u_{n} \le {\widetilde{u}}$ in $D_{i}$ , $\widetilde{u} \in L^{1}(D_{i}; V^{-} \,\mathrm {d}x)$ and $\xi \in L^{\infty }(\Omega )$, we deduce from the Dominated Convergence Theorem that

$$\begin{aligned} \lim _{j \rightarrow \infty }{\int _{D_{i}} (V^{-}u_{n_{j}} - T_{n_{j}}(V^{-}) u_{n_{j} - 1})\xi } = 0. \end{aligned}$$

As $j \rightarrow \infty$ in (7.7), we then get

$$\begin{aligned} ({\widetilde{u}}\vert \xi )_{i} = \int _{D_{i}} w_{i} h \xi \quad \text {for every }\xi \in H_{i}(\Omega ) \cap L^{\infty }(\Omega ). \end{aligned}$$

For a general $\xi \in H_{i}(\Omega )$, we apply this identity to $T_{k}(\xi )$ and then let $k \rightarrow \infty$. Equation (7.6) then follows from the density of $H_{i}(\Omega )$ in $\mathcal {H}_i(\Omega )$. Since the minimizer $\theta _{i, h}$ satisfies the same equation, it follows by uniqueness that ${\widetilde{u}} = \theta _{i, h}$. In particular, ${\widetilde{u}} = \theta _{i, h}$ almost everywhere in $D_{i}$ . $\square$

We conclude this section with an example due to Vázquez and Zuazua [28] where the space $\mathcal {H}_{i}(\Omega )$ is strictly larger than $H_{i}(\Omega )$ :

### Example 7.7

For $N \ge 3$ and $0< \alpha < N - 2$, let $u_{\alpha } : B_{1} \rightarrow {\mathbb R}$ be defined for $x \ne 0$ by

7.8 $$\begin{aligned} u_{\alpha }(x) = \frac{1}{|x|^{\alpha }} - 1 \end{aligned}$$

and take $V_{\alpha } : B_{1} \rightarrow [- \infty , 0]$ given by

7.9 $$\begin{aligned} V_{\alpha }(x) = \frac{\Delta u_{\alpha }(x)}{u_{\alpha }(x)} = - \frac{\alpha (N-2-\alpha )}{|x|^2(1-|x|^{\alpha })}. \end{aligned}$$

Then, $$u_{\alpha } \in W_{0}^{1, 1}(B_{1}) \cap L^{1}(B_{1}; V_{\alpha } \,\mathrm {d}x)$$ and

7.10 $$\begin{aligned} - \Delta u_{\alpha } + V_{\alpha } u_{\alpha } = 0 \quad \text {in the sense of distributions in }B_{1}. \end{aligned}$$

Since $S \subset \{0\}$, the set  is connected. We thus have only one completion space, which we denote by $\mathcal {H}(D)$. From [19, Example 1.2], the torsion function $\zeta _{1, \alpha }$ satisfies $\widehat{\zeta _{1, \alpha }}(0) = 0$, whence $S = \{0\}$. Since $V_{\alpha }$ is negative, $H(D) = W_{0}^{1, 2}(B_{1})$. We now verify that for $\alpha = \frac{N-2}{2}$ we have

7.11 $$\begin{aligned} u_{\alpha } \not \in H(D){} \quad \text {and} \quad {} u_{\alpha } \in \mathcal {H}(D). \end{aligned}$$

For the first assertion of (7.11), it suffices to observe that $|\nabla u_{\alpha }| = \alpha /|x|^{\alpha + 1} \not \in L^{2}(B_{1})$ when $\alpha \ge \frac{N-2}{2}$. To show that $u_{\alpha }\in \mathcal {H}(D)$ we proceed by truncation. We have

7.12 $$\begin{aligned} \int _{B_1}|\nabla T_k(u_{\alpha })|^2 = \alpha ^2 \int _{B_{1} \setminus B_{\rho _{k}}}{\frac{\,\mathrm {d}x}{|x|^{2\alpha + 2}}}, \end{aligned}$$

where $\rho _k=(k+1)^{-{1}/{\alpha }}$. Moreover, denoting by *O*(1) a quantity that is uniformly bounded with respect to *k*, we can write

7.13 $$\begin{aligned} \int _{B_{1}} V_{\alpha }T_{k}(u_{\alpha })^{2} = - \alpha (N-2-\alpha ) \int _{B_{1} \setminus B_{\rho _{k}}} \frac{\,\mathrm {d}x}{|x|^{2 \alpha + 2}} + O(1). \end{aligned}$$

When $\alpha = \frac{N-2}{2}$, the coefficients of the integrals in (7.12) and (7.13) are opposite to each other and we deduce that

$$\begin{aligned}{} \Vert T_{k}(u_{\alpha })\Vert ^{2} = \int _{B_1} \bigl ( |\nabla T_k(u_{\alpha })|^2 + V_{\alpha }T_{k}(u_{\alpha })^{2} \bigr ) = O(1). \end{aligned}$$

Hence, the sequence $(T_k(u_{\alpha }))_{k\in \mathbb {N}}$ is bounded in $\mathcal {H}(D)$. Since it converges pointwise to $u_{\alpha }$ , using Lemma 7.4 we conclude that $u_{\alpha }\in \mathcal {H}(D)$.

## Proof of Theorem 1.3

### Proof of Theorem 1.3

Let $z_{i}$ be the duality solution of

8.1 $$\begin{aligned} \left\{ \begin{alignedat}{2}&-\Delta z_{i} + V^{+} z_{i} = \mu \lfloor _{D_{i}}&\quad \text {in } \Omega ,\\&z_{i} = 0&\quad \text {on } \partial \Omega . \end{alignedat} \right. \end{aligned}$$

By (4.5), we have

8.2 $$\begin{aligned} \zeta _{Q(z_{i})} \le z_{i} \quad \text {almost everywhere in } \Omega . \end{aligned}$$

We claim that, for every $n \in {\mathbb N}$,

8.3 $$\begin{aligned} u_{n} \le u \chi _{D_{i}} \quad \text {almost everywhere in }\Omega , \end{aligned}$$

where *u* is the distributional solution of (1.1) and $(u_{n})_{n \in {\mathbb N}}$ is the sequence of duality solutions defined in Proposition 6.1 with datum $Q(z_{i})$, that is for $n \ge 1$,

$$\begin{aligned}{} \left\{ \begin{aligned}&-\Delta u_{n} + V^{+} u_{n} = T_{n}(Q(z_{i})) + T_{n}(V^{-})u_{n - 1}&\quad \text {in } \Omega ,\\&u_{n} = 0&\quad \text {on } \partial \Omega . \end{aligned} \right. \end{aligned}$$

Since for every nonnegative $f \in L^{\infty }(\Omega )$, $\zeta _{f}$ is also nonnegative, we have

8.4 $$\begin{aligned} \begin{aligned} \int _{\Omega } u_{n} f&= \int _{\Omega } \zeta _{f} (T_{n}(Q(z_{i})) + T_{n}(V^{-}) u_{n - 1})\\&\le \int _{\Omega } \zeta _{f} Q(z_{i}) + \int _{\Omega } \zeta _{f} V^{-} u_{n - 1}. \end{aligned} \end{aligned}$$

We prove (8.3) by induction. Firstly, we have $u_0 = 0 \le u \chi _{D_{i}}$ by assumption on *u*. Assume now that, for some $n \ge 1$, the inequality holds for $n - 1$. Using the duality formulation of $z_{i}$ and the induction assumption in (8.4), for every nonnegative $f \in L^{\infty }(\Omega )$ we have

$$\begin{aligned}{} \int _{\Omega } u_{n} f \le \int _{\Omega } \zeta _{Q(z_{i})} f + \int _{\Omega } \zeta _{f} V^{-} u \chi _{D_{i}}\,. \end{aligned}$$

Using (8.2) and the fact that $z_{i}$ and $u\chi _{D_{i}}$ are duality solutions of (8.1) and (1.1) with datum $\mu \lfloor _{D_{i}}$ , respectively, we then get

$$\begin{aligned} \int _{\Omega } u_{n} f \le \int _{\Omega } z_{i} f + \int _{\Omega } \zeta _{f} V^{-} u\chi _{D_{i}} = \int _{\Omega } \widehat{\zeta _{f}} \,\mathrm {d}\mu \lfloor _{D_{i}} + \int _{\Omega } \zeta _{f} V^{-} u\chi _{D_{i}} = \int _{\Omega } u\chi _{D_{i}} f. \end{aligned}$$

Since *f* is an arbitrary nonnegative function in $L^{\infty }(\Omega )$, estimate (8.3) then follows.

As a consequence of (8.3), we have

8.5 $$\begin{aligned} u_{n} = 0 \quad \text {almost everywhere in }\Omega \setminus D_{i}\,. \end{aligned}$$

Let us now show that, for every $n \ge 1$,

8.6 $$\begin{aligned} u_{n} > 0 \quad \text {almost everywhere in } D_{i}\,. \end{aligned}$$

Indeed, we observe that, by the representation formula (3.6), for almost every $x \in \Omega$ we have

$$\begin{aligned}{} z_{i}(x) = \int _{D_{i}} \widehat{G_{x}} \,\mathrm {d}\mu . \end{aligned}$$

Since $\mu (D_{i}) > 0$ and $\widehat{G_{x}} > 0$ in $D_{i}$ for $x \in D_{i}$ , we then have

$$\begin{aligned}{} z_{i} > 0 \quad \text {almost everywhere in }D_{i}\,. \end{aligned}$$

Thus, $Q(z_{i}) > 0$ almost everywhere in $D_{i}$. Applying again (3.6), for almost every $x \in \Omega$ we have

$$\begin{aligned}{} u_{n}(x) = \int _{\Omega } {G_{x}} \, (T_{n}(Q(z_{i})) + T_{n}(V^{-}) u_{n - 1}) \ge \int _{D_{i}} {G_{x}} \, T_{n}(Q(z_{i})). \end{aligned}$$

For $x \in D_{i}$ , the integrand is almost everywhere positive in $D_{i}$ and (8.6) follows.

Let $n \ge 1$. As $u_{n} \in W^{1, 2}_0(\Omega )\cap L^2(\Omega ; V^+ \,\mathrm {d}x)$ is also a variational solution for the same datum, we have

$$\begin{aligned} \int _{\Omega } (\nabla u_{n} \cdot \nabla \xi + V^{+} u_{n} \xi ) = \int _{\Omega }(T_{n}(Q(z_{i})) + T_{n}(V^-) u_{n - 1}) \xi {} \end{aligned}$$

for every $\xi \in W^{1, 2}_0(\Omega )\cap L^2(\Omega ; V^+ \,\mathrm {d}x)$. By Lemma 2.3 applied to the potential $V^{+}$ with $f = T_{n}(Q(z_{i})) + T_{n}(V^-) u_{n - 1}$ , it follows from (8.5) and (8.6) that

8.7 $$\begin{aligned} \int _{D_{i}} (|\nabla \xi |^{2} + V^{+} \xi ^{2}) \ge \int _{D_{i}} \frac{T_{n}(Q(z_{i}))+ T_{n}(V^-) u_{n - 1}}{u_{n}} \xi ^{2}, \end{aligned}$$

for every $\xi \in W^{1, 2}_0(\Omega )\cap L^2(\Omega ; V^+ \,\mathrm {d}x)$. Denote by $\widetilde{u}$ the pointwise limit of the nondecreasing sequence $(u_{n})_{n \in {\mathbb N}}$ . Since

$$\begin{aligned}{} 0 \le {\widetilde{u}} \le u \chi _{D_{i}} \in L^{1}(\Omega ), \end{aligned}$$

we have ${\widetilde{u}} < \infty$ almost everywhere in $D_{i}$ and then ${u_{n-1}}/{u_{n}} \rightarrow 1$ almost everywhere in $D_{i}$ . By Fatou’s lemma, letting $n \rightarrow \infty$ in (8.7) we obtain that

$$\begin{aligned}{} \int _{D_{i}} (|\nabla \xi |^{2} + V^{+} \xi ^{2}) \ge \int _{D_{i}} \Bigr (\frac{Q(z_{i})}{{\widetilde{u}}} + V^{-} \Bigr )\xi ^{2}. \end{aligned}$$

Hence, any measurable function $w_{i} : D_{i} \rightarrow (0, +\infty )$ such that

8.8 $$\begin{aligned} 0 < w_{i} \le \frac{Q(z_{i})}{{\widetilde{u}}} \quad \text {almost everywhere in } D_{i} \end{aligned}$$

satisfies estimate (1.8). $$\square $$

### Example 8.1

Let $u_{\alpha }$ and $V_{\alpha }$ be given by (7.8) and (7.9), respectively. We show that the assumption of Theorem 1.3 is satisfied when $\frac{N-2}{2}< \alpha < N-2$, even though (7.10) holds. Indeed, for $\frac{N-2}{2} \le \beta < \alpha$, we have

$$\begin{aligned} - \Delta u_{\beta } + V_{\alpha } u_{\beta } = f_{\alpha , \beta } \quad \text {in the sense of distributions in }B_{1}, \end{aligned}$$

where $f_{\alpha , \beta } \in L^{1}(B_{1})$ is a nonnegative function and $f_{\alpha , \beta }(x) > 0$ for $x \ne 0$. Indeed, by an explicit computation,

$$\begin{aligned} f_{\alpha , \beta }(x) = \frac{\beta (N - 2 - \beta ) (1 - |x|^{\alpha }) - \alpha (N - 2 - \alpha ) (1 - |x|^{\beta })}{|x|^{\beta + 2} (1 - |x|^{\alpha })}. \end{aligned}$$

As the function $t \mapsto t(N - 2 - t)$ is decreasing in the interval $[\frac{N-2}{2}, N-2]$, for $\beta < \alpha$ in this range we then have

$$\begin{aligned} f_{\alpha , \beta } \ge \frac{\alpha (N - 2 - \alpha )}{|x|^{\beta + 2} (1 - |x|^{\alpha })} (|x|^{\beta } - |x|^{\alpha }) > 0 \quad \text {for every }x \in B_{1} \setminus \{0\}. \end{aligned}$$

Observe that in Example 8.1 one has $u_{\beta } \not \in L^{2}(B_{1}; V^{-} \,\mathrm {d}x)$ if and only if $\beta \ge (N-2)/2$. We conclude this section showing that the expected spectral property can be recovered on each component $D_i$ provided the solution has enough integrability with respect to $V^{-}$.

### Corollary 8.2

Let $i \in I$ be such that (1.8) holds for some measurable function $w_{i} : D_{i} \rightarrow (0, +\infty )$. If *u* is a duality solution of (5.1) with $\mu \in \mathcal {M}(\Omega )$ such that $\mu \lfloor _{D_{i}}{} = 0$ and $u \in L^{2}(D_{i}; V^{-}\,\mathrm {d}x)$, then $u = 0$ almost everywhere in $D_{i}$ .

### Proof

Since $\mu \lfloor _{D_{i}}{} = 0$, by Corollary 5.3 and Proposition 5.4 the function  satisfies

$$\begin{aligned} - \Delta {\bar{u}}_{i} + V {\bar{u}}_{i} = -\tau \quad \text {in the sense of distributions in }\Omega , \end{aligned}$$

for some diffuse measure $\tau$ such that $|\tau |(\Omega \setminus S) = 0$. By [7], for every $k > 0$ we then have

$$\begin{aligned} \int _{\Omega } \bigl (|\nabla T_{k}({\bar{u}}_{i})|^{2} + V {\bar{u}}_{i} T_{k}({\bar{u}}_{i})\bigr ) = -\int _{\Omega } \widehat{T_{k}(\bar{u}_{i})} \,\mathrm {d}\tau . \end{aligned}$$

Since $|T_{k}(\bar{u}_{i})| \le |u|$ and $\widehat{u} = 0$ quasi-everywhere on *S* by Proposition 3.3, we have

$$\begin{aligned} \widehat{T_{k}(\bar{u}_{i})} = 0 \quad \text {quasi-everywhere on } S. \end{aligned}$$

Therefore, from $|\tau |(\Omega \setminus S) = 0$ it follows that

$$\begin{aligned} \int _{D_{i}} \bigl (|\nabla T_{k}(u)|^{2} + V u T_{k}(u)\bigr ) = 0. \end{aligned}$$

Using (1.8) with $T_{k}(u)$ and this identity, we get

$$\begin{aligned} \begin{aligned} \int _{D_{i}} w_{i} T_{k}(u)^{2} \le \int _{D_{i}} \bigl (|\nabla T_{k}(u)|^{2} + V T_{k}(u)^{2}\bigr )&= \int _{D_{i}} V (T_{k}(u) - u) T_{k}(u)\\&\le \int _{D_{i}} V^{-} (u - T_{k}(u)) T_{k}(u). \end{aligned} \end{aligned}$$

Since $u \in L^{2}(D_{i}; V^{-} \,\mathrm {d}x)$, by the Dominated Convergence Theorem the right-hand side converges to 0 as $k \rightarrow \infty$. Therefore, applying Fatou’s lemma we get

$$\begin{aligned} \int _{D_{i}} w_{i} u^{2} = 0. \end{aligned}$$

The conclusion follows since $w_{i} > 0$ almost everywhere in $D_{i}$ . $\square$

## Proofs of Theorems 1.2 and 1.4

### Proof of Theorem 1.2

Given $0< \alpha < 1$, we apply Theorem 1.3 with potential  and measure . We observe that

9.1 $$\begin{aligned} \nu _{\alpha }(D_{i}) > 0.{} \end{aligned}$$

This is clear when $\mu (D_{i}) > 0$. Otherwise, we have $\mu (D_{i}) = 0$ and

$$\begin{aligned}{} -\Delta u + V^{+}u = V^{-}u \quad \text {as a measure in }D_{i}. \end{aligned}$$

Applying the representation formula (3.6) and the fact that $\widehat{G_{x}} = 0$ in $\Omega \setminus D_{i}$ for $x \in D_{i}$ , we get

$$\begin{aligned}{} u(x) = \int _{D_{i}} {G_{x}} V^{-} u \quad \text {for almost every }x \in D_{i}\,. \end{aligned}$$

Since *u* is nontrivial in $D_{i}$ , the integral in the right-hand side is positive for some $x \in D_{i}$ . We then must have $\int _{D_{i}} V^{-}u \,\mathrm {d}x > 0$ and (9.1) follows.

We now let $\xi \in W_{0}^{1, 2}(\Omega ) \cap L^{2}(\Omega ; V^{+} \,\mathrm {d}x)$. Since $\nu _{\alpha }(D_{i}) > 0$, from Theorem 1.3 there exists a measurable function $w_{\alpha } : D_{i} \rightarrow (0, +\infty )$ such that

$$\begin{aligned} \int _{D_{i}} (|\nabla \xi |^2 + V_{\alpha } \xi ^{2}) \ge \int _{D_{i}} w_{i,\alpha }\xi ^2 \ge 0. \end{aligned}$$

Taking the limit as $\alpha \rightarrow 1$, by the Monotone Convergence Theorem it follows that

$$\begin{aligned} \int _{D_{i}}(|\nabla \xi |^2 + V\xi ^2) \ge 0. \end{aligned}$$

$$\square $$

### Proof of Theorem 1.4

Using the notation of the proof of Theorem 1.3, we take a bounded measurable function $w_{i} : D_{i} \rightarrow (0, +\infty )$ such that $u \in L^{2}(D_{i}; w_{i} \,\mathrm {d}x)$ and

9.2 $$\begin{aligned} 0 < w_{i} \le \frac{Q(z_{i})}{u} \quad \text {almost everywhere in }D_{i}\,. \end{aligned}$$

Since $0 < {\widetilde{u}} \le u$ almost everywhere in $D_{i}$ , this weight satisfies (8.8) and then (1.8) holds.

Denote by $(v_{n})_{n \in {\mathbb N}}$ the sequence defined in Proposition 6.1 with datum $w_{i} h \chi _{D_{i}}$ , where $h \in L^{2}(D_{i}; w_{i} \,\mathrm {d}x)$ and $0 \le h \le u$. We claim that, for every $n \in {\mathbb N}$,

9.3 $$\begin{aligned} v_{n} \le u \chi _{D_{i}} \quad \text {almost everywhere in }\Omega . \end{aligned}$$

Indeed, by (9.2) and the fact that $h \le u$ in $\Omega$,

$$\begin{aligned}{} 0 \le w_{i} h \le \frac{Q(z_{i})}{u} h \le Q(z_{i}) \quad \text {almost everywhere in }D_{i}\,. \end{aligned}$$

By comparison of solutions, we then get

$$\begin{aligned}{} v_{n} \le u_{n} \le u \chi _{D_{i}} \quad \text {almost everywhere in } \Omega , \end{aligned}$$

where $(u_{n})_{n \in {\mathbb N}}$ is the sequence used in the proof of Theorem 1.3 and the second inequality is given by (8.3).

As a consequence of (9.3), the sequence $(v_{n})_{n \in {\mathbb N}}$ is bounded in $L^{2}(D_{i}; w_{i} \,\mathrm {d}x)$ and $L^{1}(D_{i}; V^{-} \,\mathrm {d}x)$. Then, by Proposition 7.6, we have $\lim \limits _{n \rightarrow \infty }{v_{n}} = \theta _{i, h}$ almost everywhere in $D_{i}$ . To conclude, since $v_{n}$ is a duality solution we have

$$\begin{aligned}{} \int _{D_{i}} v_n f =\int _{D_i} (T_{n}(w_{i} h) + T_n(V^{-}) v_{n-1})\zeta _{f} \quad \text {for every }f \in L^{\infty }(\Omega ). \end{aligned}$$

Since $\zeta _{f}$ is bounded, $0 \le w_{i}h \le Cu \in L^{1}(D_{i})$ and $0 \le T_{n}(V^{-}) v_{n - 1} \le V^{-} u \in L^{1}(D_{i})$, it then follows from the Dominated Convergence Theorem that

$$\begin{aligned}{} \int _{D_{i}} \theta _{i, h} f =\int _{D_i} (w_{i} h + V^{-} \theta _{i, h})\zeta _{f}. \end{aligned}$$

Hence, $\theta _{i, h}\chi _{D_{i}}$ is a duality solution of (5.1) with datum $w_{i}h \chi _{D_{i}}$. As we have $\theta _{i, h} = \theta _{i, h}\chi _{D_{i}}$ in $\Omega$ and $w_{i}h + V^{-} \theta _{i, h} \in L^{1}(D_{i})$, the conclusion follows from Proposition 5.4. $\square$

The next example shows that the validity of a Poincaré inequality (1.8) is not enough to ensure that minimizers $\theta _{i, h}$ are distributional solutions of an equation of the form (1.7) for any $\mu \in \mathcal {M}(\Omega )$.

### Example 9.1

Given a smooth convex open subset $\omega \Subset \Omega$, let $V : \Omega \rightarrow [-\infty , \infty ]$ be defined on $\Omega \setminus \partial \omega$ by

$$\begin{aligned} V =\frac{1}{4d_{\partial \omega }^{2}}\big (\chi _{\Omega \setminus \omega } -\chi _{\omega }\big ) , \end{aligned}$$

where $d_{\partial \omega }$ denotes the distance to $\partial \omega$. Since *S* depends only on $V^{+}$, one shows that $S = \partial \omega$, whence $\Omega \setminus S$ has two connected components, namely  and . The Poincaré inequality (1.8) with positive weight holds for both of them. This is clear on $D_{2}$, while on $D_{1}$ there exists $\epsilon > 1/4{{\,\mathrm{diam}\,}}^{2}(\omega )$ such that

$$\begin{aligned} \int _{\omega } (|\nabla \xi |^2 + V\xi ^2) \ge \epsilon \int _{\omega } \xi ^2{} \quad \text {for every }\xi \in W_{0}^{1, 2}(\omega )\,; \end{aligned}$$

see [2, Theorem II]. Hence, for any $h \in L^{2}(D_{1})$, the functional *E* with $i = 1$ has a minimizer $\theta _{1, h}$ in $\mathcal {H}_{1}(\Omega ) \subset L^{2}(D_{1})$.

We claim that if *h* is nonnegative and $\int _{D_{1}} h > 0$, then $\theta _{1, h}$ cannot be a distributional solution of (1.1) for any $\mu \in \mathcal {M}(\Omega )$, thus extending the nonexistence of continuous supersolutions from [2, Theorem III]. To this end, it suffices to show that $V \theta _{1, h} \not \in L^{1}(D_{1})$.

Since *h* is nonnegative, we have $\theta _{1, h} \ge 0$ almost everywhere in $\omega$. Moreover,

$$\begin{aligned}{} -\Delta \theta _{1, h} - \frac{\theta _{1, h}}{4 d_{\partial \omega }^{2}} = h \quad \text {in the sense of distributions in }\omega . \end{aligned}$$

Given a nonempty open set $U \Subset \omega$ such that $\int _{U} \theta _{1, h} > 0$, let *v* be the solution of

$$\begin{aligned} \left\{ \begin{aligned}&- \Delta v = \frac{\theta _{1, h}}{4d_{\partial \omega }^{2}}\chi _{U} \quad&\text {in }\omega ,\\&v = 0 \quad&\text {on }\partial \omega . \end{aligned} \right. \end{aligned}$$

By comparison, we have $0 \le v \le \theta _{1, h}$ almost everywhere in $\omega$ ; see [23, Proposition 6.1 and Lemma 17.6]. Since *v* is a nontrivial superharmonic function in $\omega$, the Hopf lemma for *v* in a neighborhood of $\partial \omega$ implies that

$$\begin{aligned}{} \infty = \int _{\omega }\frac{v}{d_{\partial \omega }^{2}} \le \int _{\omega }\frac{\theta _{1, h}}{d_{\partial \omega }^{2}}. \end{aligned}$$

We deduce that $V \theta _{1, h} \not \in L^{1}(D_{1})$ and then $\theta _{1, h}$ cannot be a distributional solution in $\Omega$.
